# Plasma cell-free DNA promise monitoring and tissue injury assessment of COVID-19

**DOI:** 10.1007/s00438-023-02014-4

**Published:** 2023-04-14

**Authors:** Xin Jin, Yanqun Wang, Jinjin Xu, Yimin Li, Fanjun Cheng, Yuxue Luo, Haibo Zhou, Shanwen Lin, Fei Xiao, Lu Zhang, Yu Lin, Zhaoyong Zhang, Yan Jin, Fang Zheng, Wei Chen, Airu Zhu, Ye Tao, Jingxian Zhao, Tingyou Kuo, Yuming Li, Lingguo Li, Liyan Wen, Rijing Ou, Fang Li, Long Lin, Yanjun Zhang, Jing Sun, Hao Yuan, Zhen Zhuang, Haixi Sun, Zhao Chen, Jie Li, Jianfen Zhuo, Dongsheng Chen, Shengnan Zhang, Yuzhe Sun, Peilan Wei, Jinwei Yuan, Tian Xu, Huanming Yang, Jian Wang, Xun Xu, Nanshan Zhong, Yonghao Xu, Kun Sun, Jincun Zhao

**Affiliations:** 1 BGI-Shenzhen, Shenzhen, 518083 Guangdong China; 2 State Key Laboratory of Respiratory Disease, National Clinical Research Center for Respiratory Disease, Guangzhou Institute of Respiratory Health, The First Affiliated Hospital of Guangzhou Medical University, Guangzhou, 510120 Guangdong China; 3 School of Medicine, South China University of Technology, Guangzhou, 510006 Guangdong China; 4 Union Hospital, Tongji Medical College, Huazhong University of Science and Technology, Wuhan, 430022 Hubei China; 5 The Sixth Affiliated Hospital of Guangzhou Medical University, Qingyuan People’s Hospital, Qingyuan, 511500 Guangdong China; 6 Yangjiang People’s Hospital, Yangjiang, 529500 Guangdong China; 7 Department of Infectious Diseases, Guangdong Provincial Key Laboratory of Biomedical Imaging, Guangdong Provincial Engineering Research Center of Molecular Imaging, The Fifth Affiliated Hospital, Sun Yat-Sen University, Zhuhai, 519000 Guangdong Province China; 8 BGI Education Center, University of Chinese Academy of Sciences, Shenzhen, 518083 Guangdong China; 9 Institute of Cancer Research, Shenzhen Bay Laboratory, Shenzhen, 518132 China; 10 Institute of Infectious Disease, Guangzhou Eighth People’s Hospital of Guangzhou Medical University, Guangzhou, 510060 Guangdong China; 11 Guangdong Provincial Academician Workstation of BGI Synthetic Genomics, BGI-Shenzhen, Shenzhen, 518120 China; 12 Guangdong Provincial Key Laboratory of Genome Read and Write, BGI-Shenzhen, Shenzhen, 518120 China

**Keywords:** Liquid biopsy, End motif, Tissue-of-origin, COVID-19, SARS-CoV-2

## Abstract

Coronavirus 2019 (COVID-19) is a complex disease that affects billions of people worldwide. Currently, effective etiological treatment of COVID-19 is still lacking; COVID-19 also causes damages to various organs that affects therapeutics and mortality of the patients. Surveillance of the treatment responses and organ injury assessment of COVID-19 patients are of high clinical value. In this study, we investigated the characteristic fragmentation patterns and explored the potential in tissue injury assessment of plasma cell-free DNA in COVID-19 patients. Through recruitment of 37 COVID-19 patients, 32 controls and analysis of 208 blood samples upon diagnosis and during treatment, we report gross abnormalities in cfDNA of COVID-19 patients, including elevated GC content, altered molecule size and end motif patterns. More importantly, such cfDNA fragmentation characteristics reflect patient-specific physiological changes during treatment. Further analysis on cfDNA tissue-of-origin tracing reveals frequent tissue injuries in COVID-19 patients, which is supported by clinical diagnoses. Hence, our work demonstrates and extends the translational merit of cfDNA fragmentation pattern as valuable analyte for effective treatment monitoring, as well as tissue injury assessment in COVID-19.

**Supplementary Information:**

The online version contains supplementary material available at 10.1007/s00438-023-02014-4.

## Introduction

The COVID-19 pandemic has become a huge threat to global health. At present, there is still no effective etiological treatment for COVID-19, and the number of diagnosed patients increases rapidly. Currently, nucleic acid test of SARS-CoV-2, the pathogen of COVID-19, has become a standard method for diagnosis, treatment monitoring and cure (Zhang et al. 2020; Zhou et al. 2020; Zhu et al. 2020). However, many asymptomatic and discharged patients are also positive for SARS-CoV-2 test, suggesting that additional diagnostic approaches are needed for treatment monitoring of the patients. Furthermore, as is a complex disease with diverse clinical manifestation, COVID-19 causes damages to various organs including lungs, the primary infected tissue, heart, kidney, and brain; such damage could further induce organ failures, shock, acute respiratory distress syndrome and even patient mortality (Bian and Team 2020; Cheng et al. 2020; Mao et al. 2020; Remmelink et al. 2020; Andargie et al. 2021; Huang et al. 2021; Sun et al. 2021). Hence, monitoring of treatment and evaluation of organ injury could benefit the clinic, while effective and easy-to-perform approaches are still lacking currently.

Recent studies (Cheng et al. 2020; Andargie et al. 2021; Ju and Sun 2022) had reported various alterations in plasma cell-free DNA (cfDNA) of COVID-19 patients with potential translational values. Plasma cfDNA are mostly derived from dying cells and retain various cell-type-specific signatures (Jahr et al. 2001; Snyder et al. 2016; Thierry et al. 2016; Sun et al. 2018). In healthy subjects, cfDNA mostly originate from the hematopoietic system (Sun et al. 2015; Moss et al. 2018); while in various clinical scenarios, such as organ transplantation and cancer, cfDNA molecules released from the affected organs are readily detectable (Gielis et al. 2015; Otandault et al. 2019). Numerous studies have demonstrated that cfDNA is a valuable analyte for diagnosis and monitoring of various diseases (van der Pol and Mouliere 2019; Heitzer et al. 2020). CfDNA is not randomly fragmented (Ivanov et al. 2015) and its fragmentation patterns correlate with the disease status and tissue-of-origin of cfDNA therefore serves as valuable and emerging biomarkers in liquid biopsy (Lo et al. 2021; An et al. 2023). For example, Snyder et al. found that cfDNA contains nucleosome footprints that informs its tissue-of-origin (Snyder et al. 2016); Ulz et al. showed that cfDNA also contains the gene expression and transcription factor binding information (Ulz et al. 2016); Cristiano et al. developed cancer diagnosis methods based on cfDNA fragmentation pattern alone (Cristiano et al. 2019). To date, comprehensive studies on cfDNA fragmentomics in COVID-19 patients haven’t been fully explored, which may contribute to development of promising biomarkers for diagnosis and monitoring of COVID-19.

In this study, we have collected and analyzed a total of 208 blood samples from 37 COVID-19 patients and 32 controls. We report gross abnormalities, dynamics as well as organ injury signals in cfDNA, demonstrating the high clinical potential of cfDNA fragmentation pattern for disease monitoring and tissue injury assessment. In addition, our work has also proposed a feasible method to meet the urgent clinical need of better healthcare of the tremendous amount of COVID-19 patients (Siordia et al. 2020).

## Results

### Overview of the study

**Fig. 1:**
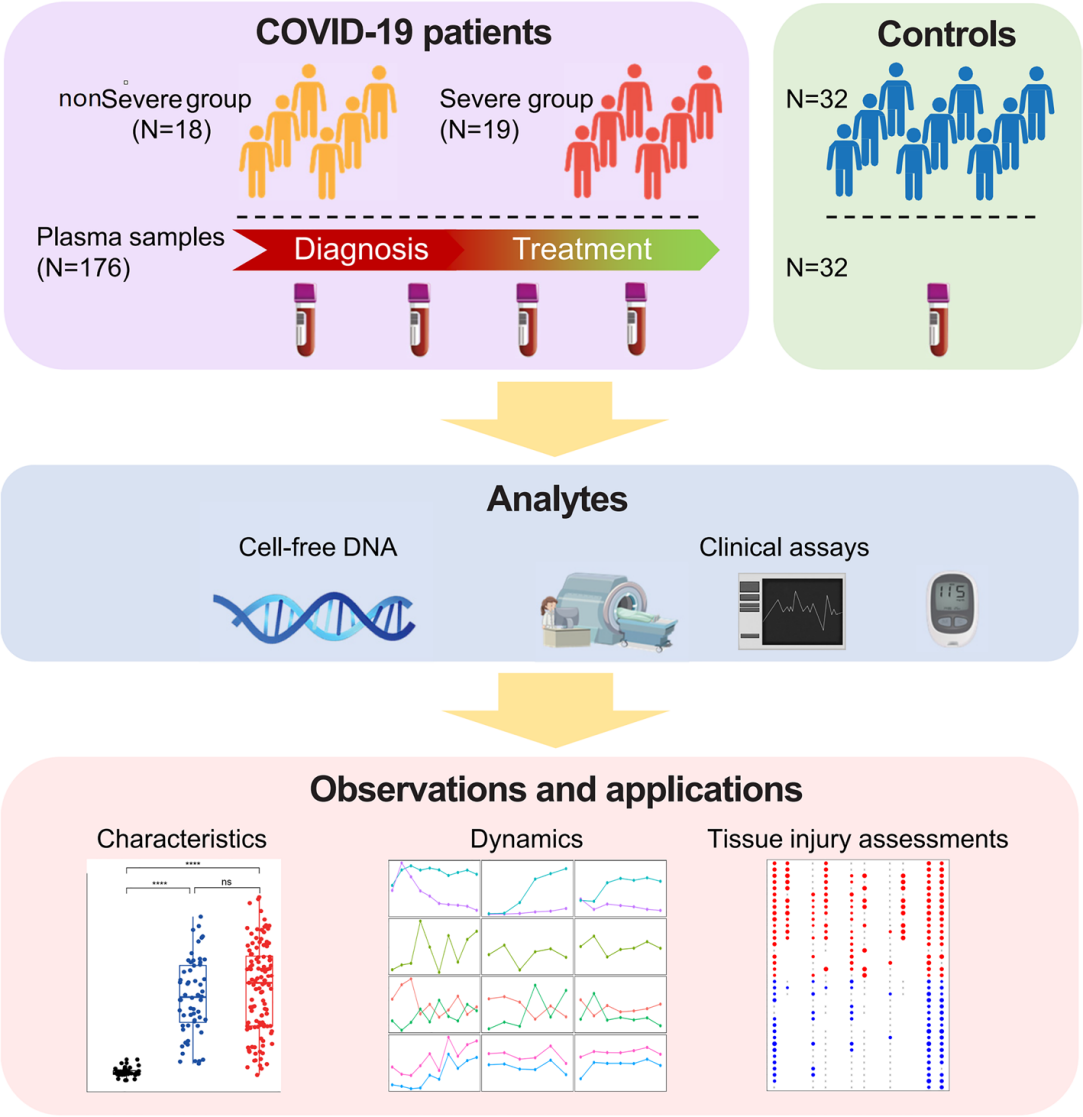
Overview of the study. A total of 37 COVID-19 patients (18 and 19 in non-severe severe conditions, respectively) and 32 healthy controls were recruited in this study. For the COVID-19 patients, 176 blood samples are collected upon hospitalization and during treatment. Plasma cfDNA is extracted and analyzed together with clinical data. As a result, we report disease-specific characteristics, dynamics, and tissue injury signals in cfDNA of COVID-19 patients

Figure 1 shows the overall design of this study. A total of 37 COVID-19 patients, either in non-severe (N = 18) or severe (N = 19) conditions, and 32 non-COVID-19 controls, were recruited from local hospitals in Guangdong province of China. Major clinical demographics of the patients could be found in Supplementary Table S1. Briefly, in the COVID-19 patients, severe cases suffer from acute severe viral pneumonia and show serious clinical symptoms that require mechanical ventilation and intensive care unit treatment, while non-severe cases show weak symptoms of pneumonia (usually minor upper respiratory tract infection) and recover within a few weeks (Docherty et al. 2020; Guan et al. 2020b; Huang et al. 2020; National Health Commission and National Administration of Traditional Chinese Medicine 2020). The controls show comparable gender distribution to COVID-19 groups, as well as comparable age and frequency of comorbidities to the non-severe group patients, while severe group patients show significantly higher age and frequency of comorbidities than controls and non-severe group. All the COVID-19 patients are immediately hospitalized upon diagnosis; for all COVID-19 patients, the first blood-collection timepoints are within 3 days after diagnosis. All COVID-19 patients receive standard treatment following the “Diagnosis and Treatment Protocol for Novel Coronavirus Pneumonia (Trial Version 5)” guidelines published by National Health Commission & National Administration of Traditional Chinese Medicine of China the therapeutic schedule had not changed over time. In short, all COVID-19 patients receive antiviral treatment; severe patients receive additional antibacterial treatment, and most of them also receive antifungal treatment. Notably, 1 severe patient also receives convalescent plasma therapy (Focosi et al. 2020; National Health Commission and National Administration of Traditional Chinese Medicine 2020) on day 16 of hospitalization. The most common comorbidity in the COVID-19 patients is hypertension (4 and 6 in non-severe and severe groups, respectively), followed by type-II diabetes. A total of 206 blood samples were collected at multiple timepoints upon hospitalization and during treatment (Supplementary Fig. S1; 2–10 samples per patient, median = 4; mostly at a 3-day interval). CfDNA from all blood samples were investigated. Key clinical data, including SARS-CoV-2-specific immunoglobulin (i.e., IgG and IgM) levels, Chest X-ray, Computed Tomography (CT) scan, coagulation profile, liver and renal functions, electrolyte, myocardial enzymes, interleukin-6, TNF-α, procalcitonin and C-reactive protein levels, were also collected (when available) during treatment to analyze the disease states of the patients (Supplementary Table S1). The plasma cfDNA was extracted, sequenced, and analyzed to investigate their correlations with COVID-19 as well as dynamics during treatment. Detailed statistics on the sequencing data are provided in Supplementary Table S2.

### Abnormalities in cfDNA of COVID-19 patients

**Fig. 2:**
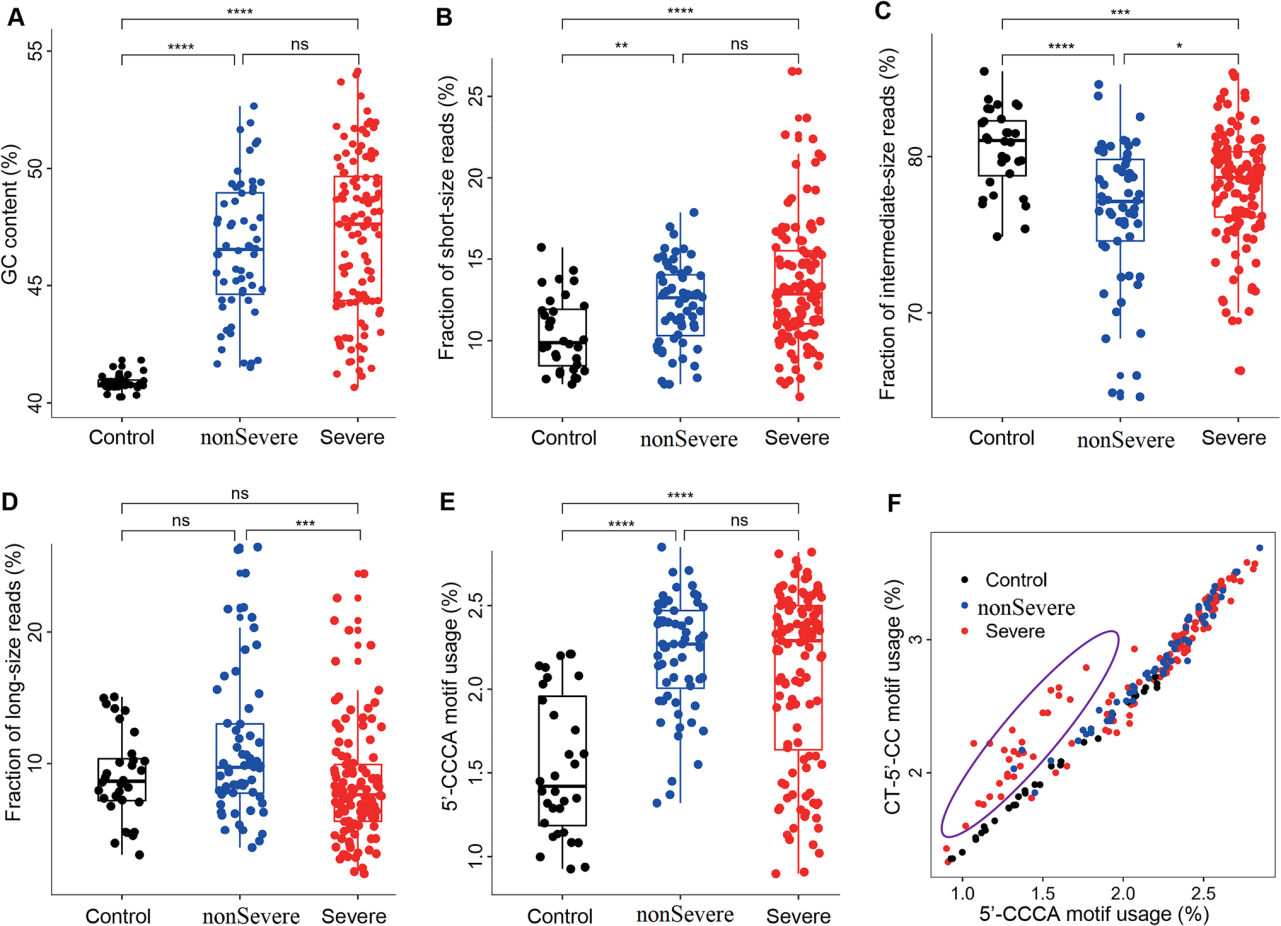
Characteristics of plasma cfDNA in COVID-19 patients. **A** GC content; **B** proportion of short (i.e., < 150 bp), **C** intermediate (i.e., 150–250 bp), and **D** long (i.e., > 250 bp) molecules; **E** proportion of reads with (i.e., usage of) 5′-CCCA end motif; **F** side-by-side comparison of 5′-CCCA and CT-5′-CC end motif usages. In panels A-E, the p-values of statistical comparisons between any groups are shown. ns: non-significant; *p < 0.05; **p < 0.01; ***p < 0.001; ****p < 0.0001

As previous studies reported that SARS-CoV-2 sequences were not detectable in plasma (Ling et al. 2020; Wolfel et al. 2020; Yang et al. 2021), we therefore focused on DNA sequences from human sources. We first investigated the global characteristics of plasma cfDNA in COVID-19 patients. Firstly, cfDNA samples from COVID-19 patients show significantly higher GC content (Fig. 2A) than controls, and the GC contents in COVID-19 patients are positively correlated with IgG levels in the peripheral blood (Supplementary Fig. S2A). Secondly, cfDNA samples from COVID-19 patients show significantly altered size patterns compared to controls. We divided the cfDNA data into short (i.e., < 150 bp), intermediate (150–250 bp), and long (i.e., > 250 bp) categories, as size pattern is a known characteristic that correlates with the tissue origin of cfDNA as well as various physiological conditions of the body (Mouliere et al. 2011; Sun et al. 2018; Han et al. 2020; Sanchez et al. 2021). As a result, cfDNA samples from COVID-19 patients show significantly higher proportions of short fragments (Fig. 2B) while lower proportion of intermediate fragments (Fig. 2C); for the proportions of long fragments, cfDNA from COVID-19 patients do not show significant differences compared to controls; however, non-severe cases show significantly increased proportion of long molecules than severe patients (Fig. 2D). Besides fragment size, end motif pattern is a newly discovered characteristic of plasma cfDNA that correlates with various physiological conditions (Serpas et al. 2019; Jiang et al. 2020). We analyzed two types of end motifs (termed as 5′-CCCA and CT-5′-CC; see “Methods” and Supplementary Fig. S2B) in our data. CfDNA samples from COVID-19 patients show significantly increased levels of 5′-CCCA and CT-5′-CC end motif usages than controls (Fig. 2E, Supplementary Fig. S2C). In addition, when 5′-CCCA and CT-5′-CC motif usages are analyzed side-by-side, the COVID-19 blood samples compose two patterns (Fig. 2F, one pattern is highlighted in purple circle). In addition, hypertension is the most common comorbidity in the COVID-19 patients; GC contents and motif usages do not show significant differences between COVID-19 patients with hypertension and without hypertension in the same group, while cfDNA size patterns show slight differences between COVID-19 patients with and without hypertension in the same group (Supplementary Fig. S3). Together, the results demonstrate gross abnormalities in cfDNA characteristics of COVID-19 patients.

### Alterations and dynamics of cfDNA characteristics during treatment

**Fig. 3:**
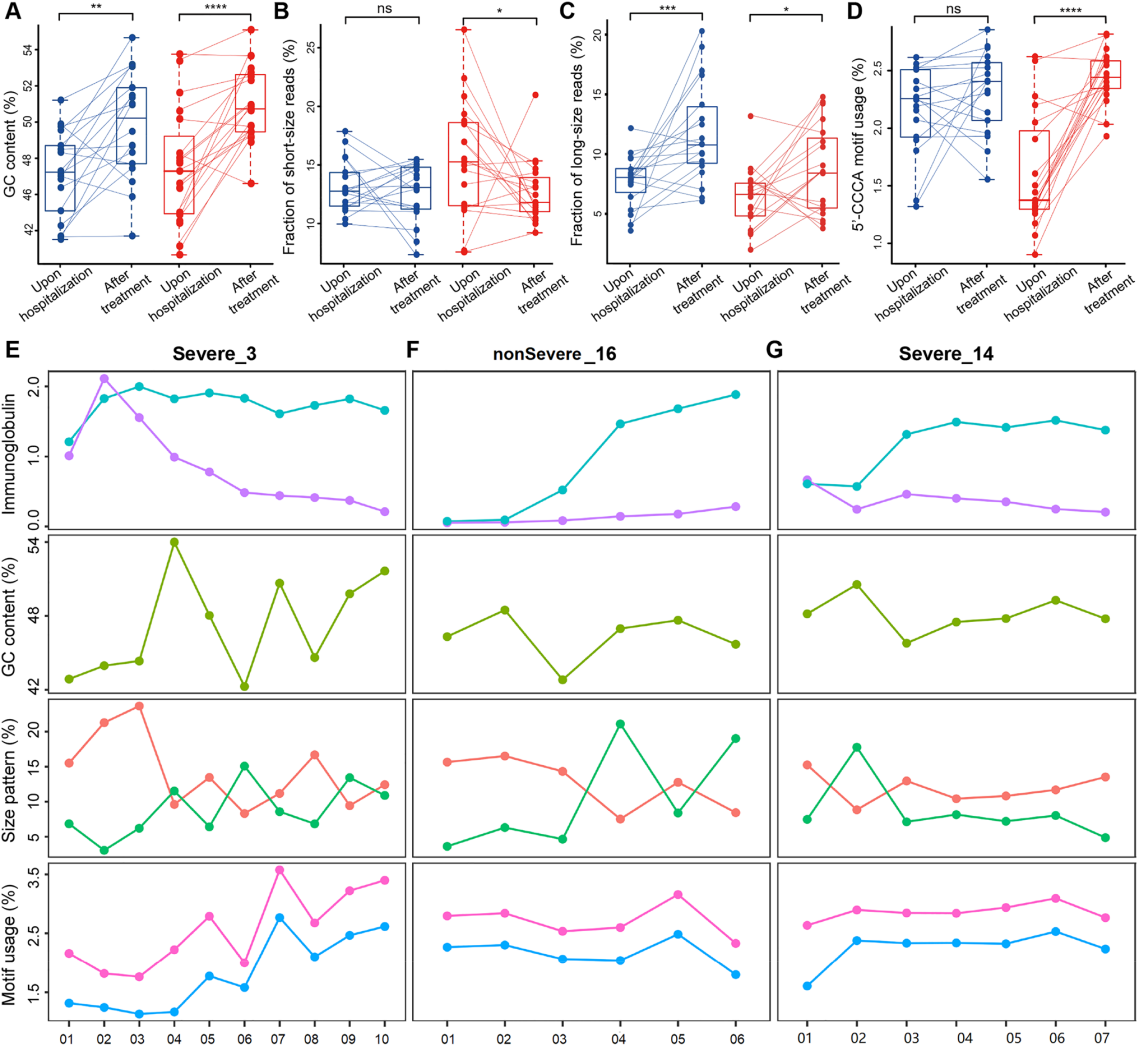
Alterations and dynamics of cfDNA characteristics in COVID-19 patients. **A**–**D** comparison of GC content, proportion of short/long reads, and usage of 5′-CCCA end motif usage between first (usually upon hospitalization) and last timepoints (when treatment has taken effect) of COVID-19 patients, respectively (dots linked by lines indicate samples from the same patients); red and blue color stand for Severe and nonSevere group, respectively. **E**–**G** SARS-CoV-2-specific immunoglobulin levels (Optical Density values), and various cfDNA characteristics during treatment of 3 representative patients. Cyan and purple lines stand for SARS-CoV-2-specific-IgG and SARS-CoV-2-specific-IgM levels, respectively; orange and green lines stand for proportion of short and long fragments, respectively; pink and blue lines stand for CT-5′-CC and 5′-CCCA end motif usages, respectively. The x-axis labels indicate the blood collection date in “Dmmdd” format; for instance, ‘D0127’ means Jan 27th, 2020. ns: non-significant; *p < 0.05; **p < 0.01; ***p < 0.001; ****p < 0.0001

We compared the plasma cfDNA characteristics at the first timepoint (i.e., upon hospitalization) versus the last timepoint, when the viral infection had come to a definition according to the “Diagnosis and Treatment Protocol for Novel Coronavirus Pneumonia (Trial Version 5)” guidelines (Fig. 3A–D, Supplementary Fig. S4). COVID-19 patients show significant increase in GC levels after treatment for both non-severe and severe groups (Fig. 3A). For cfDNA size patterns, differences in proportion of short fragments after treatment are not remarkable in non-severe patients, while significantly decreased in severe patients; in contrast, both non-severe and severe groups show significantly elevated proportion of long fragments (Fig. 3B, C). For end motif patterns, elevation in 5′-CCCA and CT-5′-CC end motif usages is marginal in non-severe patients while significant in severe patients (Fig. 3D). The results thus showed that cfDNA characteristics in COVID-19 patients change drastically during treatment.

We further investigated whether cfDNA characteristics could reflect the body responses during treatment. To do this, we profiled cfDNA characteristics along with immunoglobulin levels for COVID-19 patients over the time courses during treatment. Three representative cases (1 non-severe and 2 severe) are shown in Fig. 3E–G and the remaining cases are provided in Supplementary Fig. S5. The SARS-CoV-2-specific IgM level is an important clinical indicator for effective immune response to SARS-CoV-2 infection (Wang et al. 2020b; Xu et al. 2020; Zhu et al. 2020). Hence, for the patient shown in Fig. 3E, the immune system starts to take effect from the second timepoint, when SARS-CoV-2-specific IgG level also starts to increase; however, the other 2 cases (Fig. 3F, G) do not show convincing SARS-CoV-2-specific IgM signal, suggesting possible immune deficiency or insufficient immunization. CfDNA characteristics also show dynamics during treatment in these samples, such as the proportion of long fragments at certain timepoints. In particular, cfDNA end motif patterns gradually increase in the patient shown in Fig. 3E while remain modestly changed in the other two cases.

### Tissue injury signals in cell-free DNA

**Fig. 4:**
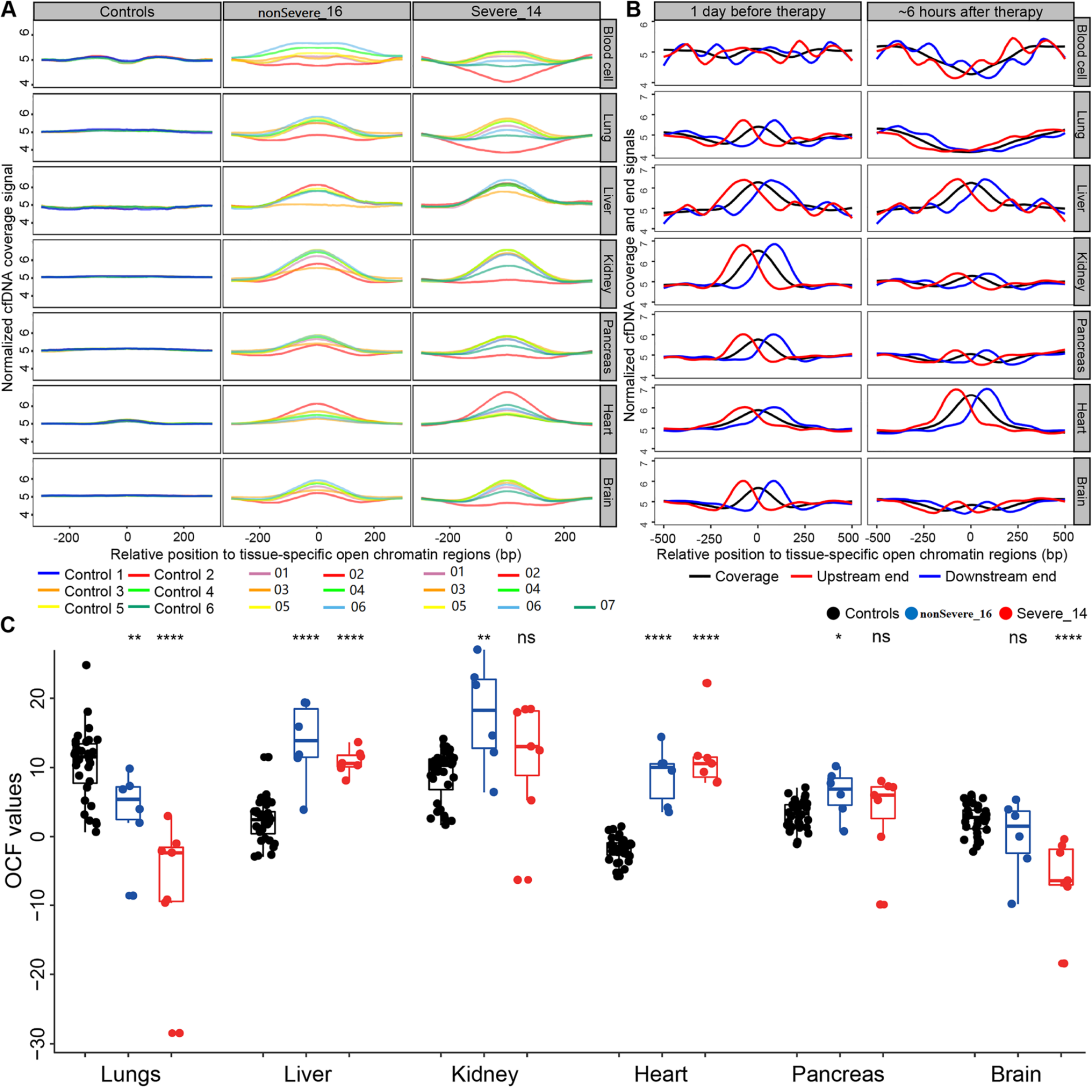
CfDNA fragmentation patterns around tissue-specific open chromatin regions. **A** normalized cfDNA coverage around tissue-specific open chromatin regions in controls, representative non-severe and severe cases, respectively. For controls, all samples are plotted, and each color represent 1 patient; for COVID-19 patients, colors represent different sample collection timepoints. Each row present one tissue and the y-axis show the normalized cfDNA coverage. **B** Plasma cfDNA from 1 day before, and ~ 6 h after treatment of a patient receiving convalescent plasma therapy. Each row present one tissue; y-axis present the normalized read coverage (black line) and orientation-aware end signals (red and blue lines). **C** comparison of OCF values between controls and two representative COVID-19 patients. OCF (Orientation-aware CfDNA Fragmentation) is a measurement approach of cfDNA fragmentation pattern as defined in our previous work (Sun et al. 2019). Each tissue-of-interest has 3 columns: black, blue, and red dot represents one control, one timepoint in the non-severe case, and one timepoint in the severe case, respectively. The “ns” and asterisks represent the statistical comparisons between the COVID-19 cases and controls. ns: non-significant; *p < 0.05; **p < 0.01; ***p < 0.001; ****p < 0.0001

To explore whether plasma cfDNA could reflect organ damages induced by COVID-19, we adapted our previous orientation-aware cfDNA fragmentation analysis approach (Sun et al. 2019) to detect signals linked to the tissue origins of cfDNA. Notably, besides blood cells, we focused on lungs, liver, heart, kidneys, pancreas, and brain in this study (Supplementary Tables S3–S5), because these organs are known to be infected by SARS-CoV-2 (Bian and Team 2020; Cheng et al. 2020). CfDNA fragmentation patterns for controls are consistent with our previous report that cfDNA coverage decreases in the tissue-specific open chromatin regions if the corresponding tissues contribute DNA in plasma (e.g., blood cells; Fig. 4A), as nucleosome-depletion in such regions makes the DNA unprotected from nuclease digestion (Sun et al. 2019); however, we find that cfDNA coverage in the open chromatin regions increase in most COVID-19 samples (Supplementary Fig. S6), which may be due to the elevated GC content in cfDNA of COVID-19 patients, as GC content for tissue-specific open chromatin regions are higher than adjacent regions (Supplementary Fig. S7); nevertheless, altered fragmentation signals (e.g., imbalanced coverage patterns) around tissue-specific open chromatin regions are still observed in certain timepoints in almost all severe COVID-19 patients. Figure 4A shows the coverage signal from the same patients as Fig. 3F–G, and remaining cases are provided in Supplementary Fig. S6. For instance, strong fragmentation signals around lung-, pancreas- and brain-specific open chromatin regions are observed at timepoint 2 of the severe case, which echoes the altered cfDNA characteristics (e.g., increase of long fragments) of this patient at the same timepoint (Fig. 3G).

As an interesting example, we investigated the severe patient who receives convalescent plasma therapy (Focosi et al. 2020; National Health Commission and National Administration of Traditional Chinese Medicine 2020) on day 16 of hospitalization. Blood samples are taken 1 day before and ~ 6 h after treatment. Both GC content, size and end motif patterns change remarkably after treatment (Supplementary Table S1). Orientation-aware cfDNA fragmentation analysis reveals drastic signal changes after treatment: both coverage and ends around blood cell-, lung-, kidney-, and brain-specific open chromatin regions alter sharply (Fig. 4B). Indeed, clinical records of this patient show various positive changes after treatment that are related to these organs, including returning to normal body temperature and improvements in the lung condition (lesions in the lower right lung field are slightly reduced according to chest radiograph and relief of respiratory distress) as well as consciousness state (increased dose of sedative and muscle relaxant).

**Fig. 5:**
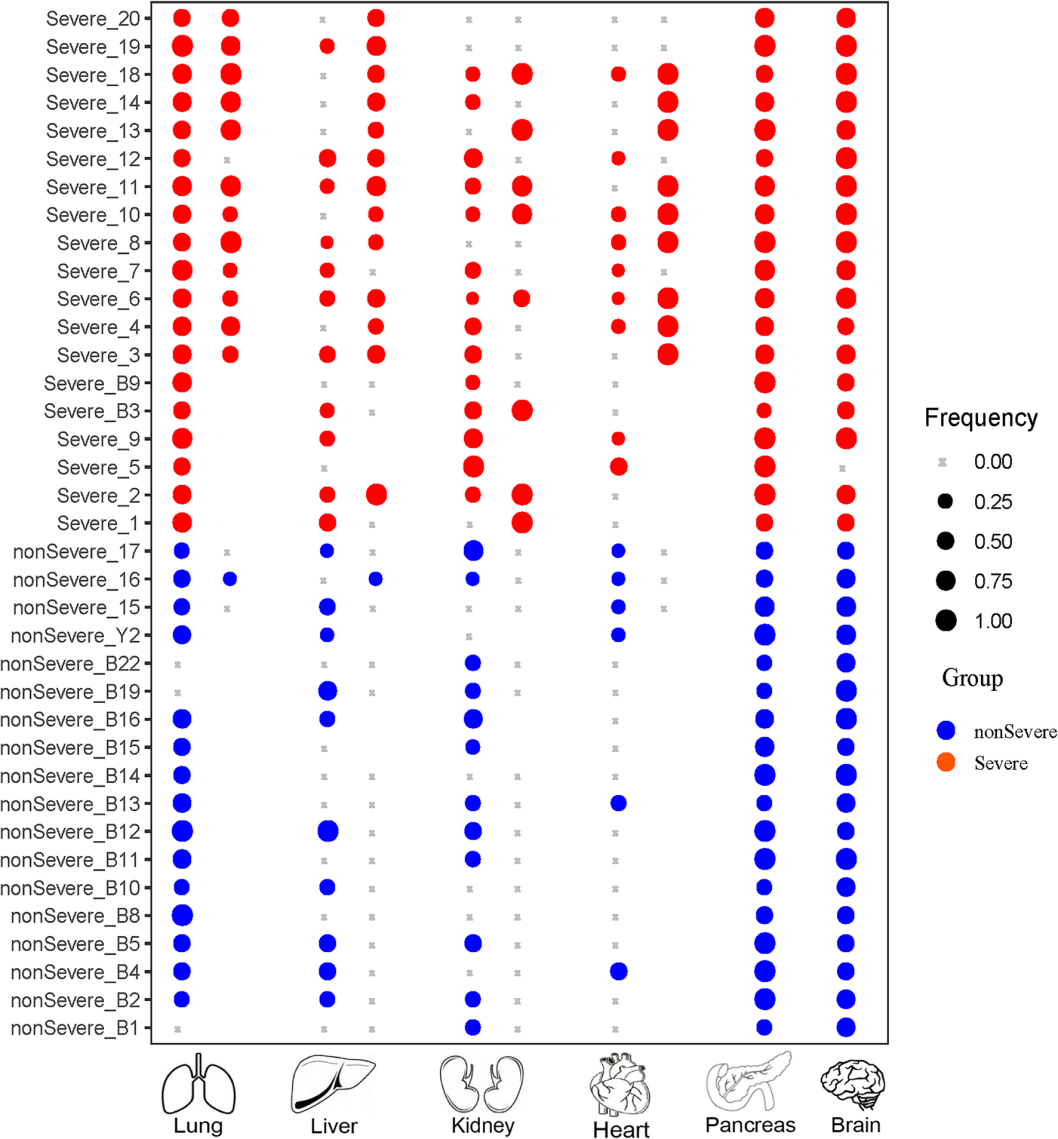
Summary of tissue injury assessment in all COVID-19 patients. For lungs, liver, kidney, and heart, the two columns represent frequencies of cfDNA samples that are predicted to suffer from injuries based on cfDNA fragmentation pattern analysis (left) and clinical diagnoses (right), respectively, for each patient. Blank points mean that clinical diagnoses are not available for these patients. For pancreas and brain, clinical diagnoses are not available for all patients and only the results from cfDNA fragmentation pattern analysis are shown

Moreover, cfDNA fragmentation patterns for lungs, liver, heart, kidney, pancreas, and brain were quantified using our previous OCF (Orientation-aware CfDNA Fragmentation) approach (see “Methods”) (Sun et al. 2019). The results for the two presentative patients illustrated in Fig. 3F, G are shown in Fig. 4C, and the results for the rest patients are shown in Supplementary Fig. S8. In general, significantly altered OCF values are observed in the majority of patients and/or tissues, suggesting prevalent tissue injuries in COVID-19 patients. Notably, in COVID-19 patients, OCF values are decreased for lungs and brain, while they are elevated for other tissues. We also observe abnormal OCF values in certain timepoints in the COVID-19 patients while the overall statistical comparisons do not show significant differences (mostly due to limited number of timepoints in this patient or other timepoints show similar OCF values to the controls). To overcome this drawback and to provide explicit tissue injury assessment results, we further built a machine learning-based classification model to predict the tissue injuries based on the orientation-aware cfDNA fragmentation signals (see “Methods”). The results are summarized in Fig. 5. Notably, clinical diagnoses on tissue injuries for lungs, liver, kidneys, and heart are also available for a proportion of patients. Frequent injuries are observed in various tissues, including lungs, pancreas, and brain, which results are consistent with clinical diagnoses for the majority of patients.

## Discussion

The outbreak of COVID-19 has last for several years. Considering the unclear therapeutics, disease monitoring is of high clinical value for better management and healthcare of the large amount of COVID-19 patients; however, efficient methods are still limited, especially for assessment of various organ injuries (Wang et al. 2020a). In this proof-of-principle study, we have conducted a comprehensive analysis of 208 blood samples collected from 37 COVID-19 patients and 32 controls. We had revealed gross abnormalities and dynamics in a broad range of cfDNA fragmentation patterns. We reported increased GC content, altered size and end motif patterns (Fig. 2A–D), which extended previous studies on elevated cfDNA concentration and neutrophil extracellular traps (NETs) in COVID-19 (Ng et al. 2020; Thierry and Roch 2020; Zuo et al. 2020; Hammad et al. 2021). COVID-19 patients suffer from active immune response to the viral infection and produces high level of immunoglobulins (Sewell et al. 2020; Wang et al. 2020b), which prefer binding/protecting GC-rich DNA (e.g., DNA molecules originated from the open chromatin regions) (Uccellini et al. 2012), suggesting that immune response may be responsible to the abnormalities in plasma cfDNA characteristics in COVID-19 patients. However, more efforts are needed to elucidate the underline biological mechanisms (e.g., chromatin status, replication timing, etc.). Moreover, the NET process is known to generate long cfDNA molecules; Fig. 2D shows that non-severe COVID-19 patients tend to have increased long cfDNA molecules than controls while severe patients do not, suggesting that patients in the non-severe group may have a higher innate immune activity than those in the severe group, which is consistent with their weaker symptoms. In the meantime, Fig. 3C shows that after treatment, the proportions of long cfDNA molecules are increased in both non-severe and severe COVID-19 patients, suggesting more NETs, i.e., enhanced immune responses of the patients, after treatment. It is also interesting to see differences in size patterns between COVID-19 patients with and without hypertension (Supplementary Fig. S3), as previous studies have demonstrated that cfDNA alterations could serve as a diagnostic biomarker for cardiovascular diseases (Polina et al. 2020). In addition, end motif analysis reveals two patterns in COVID-19 patients; interestingly, most of the samples that form the altered pattern (Fig. 2F, purple circle) are collected at the first or second timepoints of severe patients, when the patients’ conditions are most critical (e.g., in a coma). Plasma cfDNA fragmentation patterns could be affected by various biological and clinical scenarios, while current knowledge is still limited. Hence, the altered cfDNA signals may suggest aberrant, yet elusive, cell death in COVID-19 patients.

Furthermore, cfDNA reveal disease dynamics and organ injury signals during the treatment. For instance, significant changes are observed in cfDNA samples at the last timepoint compared to the first timepoint (Fig. 3A–D), indicating that cfDNA characteristics could reflect therapeutic efficacies. Moreover, cfDNA show fragmentation signals around tissue-specific open chromatin regions in various cases, which is partly in line with clinical observations on organ injuries in these patients. In fact, organ injury in COVID-19 patients may correlate and partially explain the altered characteristics in cfDNA, as cells in damaged organs may die abnormally thus release DNA with aberrant fragmentation patterns (Fig. 2) (Higuchi 2003). As an interesting example, cfDNA from a severe case receiving plasma therapy show huge alterations ~ 6 h after treatment: we observe drastic changes around blood cell- and lung-specific open chromatin regions, suggesting that the patient has responded to the treatment, especially the lungs, which is evidenced by the clinical observations; kidney-specific open chromatin regions also show strong fragmentation patterns after treatment, which is reasonable because kidney is an important organ for metabolism and is known to involve in COVID-19 (Ronco and Reis 2020). Hence, the data indicate that cfDNA analysis is indeed sensitive in monitoring the body response during treatment. The mechanism beneath aberrant size patterns of cfDNA in COVID-19 patients is unclear and may related to various factors, such as inflammation, neutrophil extracellular traps (NETs) (Thierry and Roch 2020), necrosis, and treatment.

Detection and monitoring of organ injuries are highly valuable for various diseases. For COVID-19, tissue injury assessment could be indicative for potential sequelae as COVID-19 patients frequently suffer from multiple tissue injuries even months after discharge (Huang et al. 2021), and organ failure is a major cause of mortality in COVID-19 (Epidemiology Working Group for Ncip Epidemic Response and Prevention 2020; Huang et al. 2020). In this study, we quantified orientation-aware fragmentation patterns (i.e., OCF values) and compared them between COVID-19 patients and controls (Fig. 4C, and Supplementary Fig. S8); our results suggest that tissue injuries are indeed common in COVID-19 patients. Interestingly, the OCF values for lungs and brain show an opposite direction in COVID-19 patients compared to other tissues. The underline mechanisms are elusive; while for lungs, we think that it may be related to their unique position as the primary organ of viral infection where frequent non-apoptotic cell deaths may occur. Besides the statistical comparisons, we further developed a machine learning-based approach to for qualitative (i.e., yes or no) measurement of tissue injuries, which could provide an explicit result for easier interpretation of the data.

Considering that cfDNA analysis could reveal tumor signals long before clinical diagnosis (Chan et al. 2017), which may partially explain the differences in cfDNA analysis and clinical diagnoses. In fact, cfDNA analysis shows that almost all COVID-19 patients suffer from lung injury which is consistent with the fact that lungs are the primary infection sites in COVID-19. Besides lungs, kidneys, pancreas, and brain are other organ with frequent injuries, which is consistent with clinical reports on COVID-19 (Aloysius et al. 2020; Khatoon et al. 2020; Naicker et al. 2020). Hence, COVID-19 induced low-level oxygen in the blood, blood clots, and cytokine storms can cause kidneys to malfunction (Batlle et al. 2020); diabetes is one of the most common comorbidities in COVID-19 patients and COVID-19 also causes diabetic symptoms in the non-diabetic patients (Guan et al. 2020a; Rubino et al. 2020); neurological abnormalities are also common in COVID-19 patients (Antony and Haneef 2020; Helms et al. 2020). In particular, the dynamics of cfDNA characters and tissue injury signal for a non-severe and a severe patient (Figs. 3, 4) show favorable consistency (e.g., kidney injury signal in the non-severe case, and signals of multiple tissue injuries in the severe case), demonstrating the potential of cfDNA fragmentation patterns in treatment monitoring and tissue injury assessment.

There are several limitations in this study that could be addressed in future research. Firstly, we could not collect any pre-treatment samples from the COVID-19 patients during the pandemic, which disabled us to explore the alterations in cell-free DNA characteristics that are caused by the disease while not confounded by treatment. Secondly, clinical data for tissue injury assessment is incomplete in this study as it usually requires dedicated assays of each tissue, while such assays may not be feasible, or with a low priority, when the medical system is overloaded during the outbreak of the pandemic. As a contrast, cfDNA is much more favorable as it able to profile the injury landscape of various organs from one tube of peripheral blood with low experimental complexity, therefore promises a more efficient and convenient approach for such task. However, the definition of “tissue injury” could be different between cfDNA analysis and clinical settings: a tissue is considered as injured in cfDNA analysis if one can detect its signal in plasma (which means cells are dying in this tissue), while in clinical, levels of hallmark proteins (could be actively released by the cells) are used as determents of tissue injury. The result on heart tissue in Fig. 5 was a typical example: cfDNA analysis did not show frequent injuries in COVID-19 patients (as cardiocytes regenerate in a very low frequency) while clinical data did. Hence, we think that cfDNA analysis might serve as a “supplementary”, while not “replacement”, to clinical assays in terms of tissue injury assessments. On the other hand, we need to point out that we could only perform qualitative analyses for tissue injuries in the current study. Hence, it would be favorable to explore the feasibility of other analyses, such as nucleosome positioning (Snyder et al. 2016; Sun et al. 2019) and promoter coverage patterns (Ulz et al. 2016), for quantitative measurement of organ injuries in future works.

As a summary, using COVID-19 as a model, we report gross alterations, patient-specific dynamics during treatment, as well as organ-specific signals in cfDNA fragmentation patterns, demonstrating that cell-free DNA fragmentation patterns could serve as valuable analytes for effective disease monitoring and tissue injury assessment in non-cancerous diseases, thus extends the applicable clinical scenarios of cfDNA in liquid biopsy, especially for the COVID-19 pandemic.

## Methods

### Ethics approval and patient recruitment

This study had been approved by The First Affiliate Hospital of Guangzhou Medical University Ethics Committee, and the institutional review board of BGI; written informed consents had been obtained from all patients and healthy donor participated in this study. A total of 37 COVID-19 patients and 32 non-COVID-19 controls were recruited from local hospitals in Guangdong. The COVID-19 patients were divided into non-severe (N = 18) or severe (N = 19) groups according to the Guidelines for COVID-19 Diagnosis and Treatment (Trial Version 5) (National Health Commission and National Administration of Traditional Chinese Medicine 2020) issued by the National Health Commission of China. Control subjects were collected from the same hospitals as the COVID-19 patients based on the following criteria: negative for SARS-CoV-2 tests on the blood-taken day and has never been diagnosed to have COVID-19 until the end of this study, and comparable age distribution to the COVID-19 patients. Blood samples were collected during Jan 27 to Mar 28, 2020 (Supplementary Table S1).

### Clinical data acquisition and analysis

The epidemiological, demographic, clinical, laboratory characteristics and treatment data were extracted from electronic medical records, and all the data had been double-checked by the relevant physicians to ensure the accuracy and completeness of the epidemiological and clinical findings. Frequency of clinical examinations was determined by the physicians-in-charge. Summarized statistics and detailed clinical information could be found in Supplementary Table S1 and S2, respectively.

Diagnoses of severe pneumonia and ARDS (Acute Respiratory Distress Syndrome) in the COVID-19 patients were according to Diagnosis and Treatment Protocol for Novel Coronavirus Pneumonia (Trial Version 5) (National Health Commission and National Administration of Traditional Chinese Medicine 2020) and the Berlin Definition (Force et al. 2012), respectively. Kidney injury was diagnosed according to the Kidney Disease: Improving Global Outcomes (KDIGO) guideline (Khwaja 2012). Heart injury was diagnosed if serum levels of cardiac biomarkers (e.g., cardiac troponin I) were above the 99th percentile upper reference limit, or if new abnormalities were shown in electrocardiography and echocardiography (Huang et al. 2020). Liver function indicators measured on admission, including alanine aminotransferase (ALT), aspartate aminotransferase (AST), direct bilirubin, etc.; patients whose ALT or AST is above the normal range were considered to suffer from liver function abnormality (Chen et al. 2020a, b). Pancreatic function tests were not carried out for most patients in our cohort; in addition, most patients are in a state of sedation and neurologic examinations (e.g., brain MRI) were also omitted (Helms et al. 2020).

### CfDNA extraction and processing

All blood samples (including those from the controls and COVID-19 patients) are collected and processed according to consensus guideline for cell-free DNA analysis (Meddeb et al. 2019). Briefly, for each sample, 1 ml peripheral blood was collected using EDTA anticoagulant-coated tubes, then centrifuged at 1600 g for 10 min at 4 °C within six hours after collection; the plasma portion was harvested and recentrifuged at 16,000 g for 10 min at 4 °C and to remove blood cells. Cell-free DNA was extracted from 200 µl plasma using MagPure Circulating DNA KF Kit (MD5432-02, Magen) following the manufacturers’ protocols. Sequencing libraries was prepared using MGIEasy Cell-free DNA Library Prep kit (MGI) on the amplified cfDNA following the manufacturer’s protocol. All the cfDNA libraries passed quality control and sequenced on DNBSEQ platform (BGI) in paired-end 100 bp mode. Statistics on sequencing data are provided in Supplementary Table S2.

### CfDNA sequencing and data processing

We used SOAPnuke (v1.5.0) (Chen et al. 2018) software to trim sequencing adapters, filter low quality and high ratio Ns in the raw reads with default parameters. The preprocessed reads were then aligned to the human reference genome (NCBI build GRCh38) using BWA (Li and Durbin 2009) software with default parameters. After alignment, PCR duplicates were removed using in-house programs: if more than two reads shared the same start and end positions, only one was kept for following analyses and the others were discarded as PCR duplicates.

### CfDNA characteristics profiling

For each cfDNA sample, GC content was determined as the proportion of G or C in the sequenced nucleotides; fragment size for each molecule was determined as the distance between the two outmost ends obtained from the alignment result; short fragments were defined as reads shorter than 150 bp, and long fragments were defined as reads longer than 250 bp. Considering that most nucleases in mammals function in an endonuclease manner (i.e., they bind to DNA and cut within the bound sequence), besides the 4-mer motifs at the 5′-end of cfDNA (i.e., 5′-CCCA) analyzed in previous studies (Serpas et al. 2019; Jiang et al. 2020), in this study, we proposed a novel 4-mer motif definition, CT-5′-CC, which extended 2 bp upstream from the 5′-end. In fact, a recent study had demonstrated that cfDNA end motifs with extensions of the 5′-end showed high accuracy in lung cancer diagnosis (Guo et al. 2022). 5′-CCCA motif usage was calculated as the proportion of reads starting with CCCA, and CT-5′-CC motif usage was calculated as the proportion of reads starting with CC and the 2 bp in the genome prior to the 5′-end are CT. The definition of 5′-CCCA and CT-5′-CC motifs are illustrated in Supplementary Fig. S2B. As a result, the previous definition presents CCCA while our new definition reveals CTCC as the motif with the highest usage. Notably, in our cohort, the CT-5′-CC motif usage is positively correlated with, and always higher than, 5′-CCCA, suggesting that our newly discovered CT-5′-CC motif could also reflect enzymatic preferences during cell apoptosis.

### Orientation-aware cfDNA fragmentation analysis

In our previous work (Sun et al. 2019), we had mined and investigated tissue-specific open chromatin regions for blood cells, lungs, liver, intestines, breast, ovary, and placenta. Based on clinical reports on tissue injuries of COVID-19 patients (Bian and Team 2020), we added kidney, pancreas, heart, and brain into the tissue list, while removed placenta from the tissue list (as there is no pregnancy samples in our cohort) in the current study. Tissue-specific open chromatin regions for all the tissues in the list were mined using the same algorithm as described in our previous work. The accession numbers of the Dnase I hypersensitivity data and the final list of tissue-specific open chromatin regions used in this study were summarized in Supplementary Table S4. For each cfDNA sample, coverage and end pattern around the tissue-specific open chromatin regions were profiled using the same algorithm as described in our previous work (Sun et al. 2019). To minimize the biases of the abnormally high coverage in the center of open chromatin regions in COVID-19 patients (Fig. 4A), OCF values for each patient and tissue were quantified using (− 210, − 180) and (180, 210) windows around the tissue-specific open chromatin regions.

### Prediction of tissue injury using cfDNA fragmentation pattern

Considering that the GC content is significantly elevated in COVID-19 samples (Fig. 2A), to minimize the potential biases (e.g., from sequencing), we developed a new method to infer tissue injury signals that solely depends on the cfDNA data from the COVID-19 samples. Based on the knowledge that blood cells are the major contributor of cfDNA in most clinical scenarios (Lui et al. 2002; Sun et al. 2015) and to date there is no clinical/genetic evidence of ovary injuries in COVID-19 patients (in fact, a large proportion of the COVID-19 patients are male in our cohort), we utilized the orientation-aware cfDNA fragmentation pattern around blood cell- and ovary-specific open chromatin regions from all COVID-19 blood samples as positive and negative signals, respectively, to train a classification model for injury assessment of other tissues. Briefly, for each cfDNA sample, after profiling of orientation-aware cfDNA end signals around the tissue-specific open chromatin regions, for all the tissues-of-interest (i.e., blood cell, ovary, lungs, liver, kidneys, pancreas, heart, and brain), the differences in normalized upstream (*U)* and downstream (*D)* end signals were calculated for each locus in two symmetrical 30 bp windows around the corresponding tissue-specific open chromatin regions (i.e., (− 210, − 180) and (180, 210)); hence, a vector of 60 values would be obtained for each tissue; then, we collected all the vectors for blood cells and ovary in the COVID-19 blood samples as positive and negative datasets, respectively, to train a classification model using SVM (Support Vector Machine) approach (Chang and Lin 2011). During training, a fivefold cross-validation was employed, which showed an overall accuracy of 93.5% on the training dataset. After model-training, for each of the tissue-of-interest, we applied the SVM classification model on its *U* and *D* end signal difference vector to determine whether it showed injury or not, during which procedure a score (calculated by the classification model) of 0.8 was used as the classification cutoff. Lastly, for each patient, we calculated the frequency of positive injury predictions in his/her blood samples for all the tissues-of-interest as the final prediction results (Fig. 5).

### Statistical analysis

Comparisons of cfDNA characteristics between COVID-19 patients and controls were performed using Mann–Whitney *U* test; comparisons of cfDNA characteristics for COVID-19 patients at the first and last timepoint were conducted using Wilcoxon signed-rank test; comparisons between OCF values for COVID-19 patients and controls were performed using Mann–Whitney *U* test. All p-values are two-tailed and a p-value lower than 0.05 was considered as statistically significant.

### Data access

The data that support the findings of this study have been deposited into CNGB Sequence Archive (CNSA) (Guo et al. 2020) of China National GeneBank DataBase (CNGBdb) (Chen et al. 2020a; b) with accession number CNP0001306 (https://db.cngb.org/cnsa/project/CNP0001306_ba039637/reviewlink/).

## Supplementary Information

Below is the link to the electronic supplementary material.

Supplementary file1 Table S1. Statistics and key clinical data of the COVID-19 patients, including immunoglobulin levels, blood routine and biochemistry, infection-related biomarkers, and coagulation function results. Statistical comparisons for each factor between controls and COVID-19 patients (XLSX 87 KB)

Supplementary file2 Table S2. Statistics of the cfDNA data, and cfDNA analysis results for each sample (XLSX 38 KB)

Supplementary file3 Table S3. Clinical evidence of organ injury for all COVID-19 patients (XLSX 86 KB)

Supplementary file4 Table S4. Tissue-specific open chromatin regions used in this study (XLSX 3655 KB)

Supplementary file5 Table S5. Summarizing the predictions for the tissue injury signals in cell-free DNA (XLSX 10 KB)

Supplementary file6 Fig. S1. Blood sample collection timepoints of COVID-19 patients in cohort 1 (PDF 192 KB)

Supplementary file7 Fig. S2. CfDNA characteristics in COVID-19 patients. (a) correlation between GC content in cfDNA of COVID-19 patients and SARS-CoV-2-specific IgG level (measured as OD values) in the corresponding blood sample; (b) definition of cfDNA end motifs used in this study; (c) Proportion of reads with CT-5′-CC end motif between controls and COVID-19 patients in cohort 1 and (d) cohort 2; (e) Clustering result of all the cfDNA samples in cohort 1 based on multiple characteristics (yield, GC content, size pattern, and 5′-CCCA end motif usage) (TIF 358 KB)

Supplementary file8 Fig. S3. Dynamics of cfDNA characteristics during treatment of COVID-19 patients. (a-b) cfDNA concentration and CT-5′-CC end motif usage at the first timepoint versus the last timepoint in cohort 1; (c-d) cfDNA concentration and CT-5′-CC end motif usage at the first timepoint versus the last timepoint in cohort 2. For each panel, the 5 columns represent controls, first and last timepoints for nonSevere (blue) and severe (red) patients, respectively (PDF 1067 KB)

Supplementary file9 Fig. S4. Time-series analysis of immunoglobulin levels and cfDNA characteristics for all COVID-19 patients in cohort 1. SARS-CoV-2-specific immunoglobulin levels are missing in some samples and are shown in blank. CfDNA concentration is measured as ng/ml; orange and green lines stand for proportion of short and long fragments, respectively; purple and blue lines stand for CT-5′-CC and 5′-CCCA end motif usages, respectively (TIF 273 KB)

Supplementary file10 Fig. S5. CfDNA coverage signal around tissue-specific open chromatin regions in multiple timepoints during treatment for COVID-19 patients in corhort 1 (PDF 662 KB)

Supplementary file11 Fig. S6. CfDNA coverage signal around tissue-specific open chromatin regions for COVID-19 patients in cohort 2 (PDF 14562 KB)

Supplementary file12 Fig. S7. GC content around tissue-specific open chromatin regions (TIF 447 KB)

Supplementary file13 Fig. S8. Summary of predicted tissue injuries in all COVID-19 patients in cohort 2 based on orientation-aware cfDNA fragmentation pattern analysis. For each case, tissue injuries of lungs, liver, kidneys, pancreas, heart, and brain are predicted. Clinical diagnoses were not available for all the patients in this cohort (PDF 281 KB)

## Data Availability

Data are available online at http://db.cngb.org/cnsa/project/CNP0001306_ba039637/reviewlink/.
