# Supplementary figures and images for: Plasma cell-free DNA promise monitoring and tissue injury assessment of COVID-19
Source: Mol Genet Genomics. 2023 Apr 14;298(4):823–36. doi: 10.1007/s00438-023-02014-4 (PMC10104435; doi:10.1007/s00438-023-02014-4)

Severe

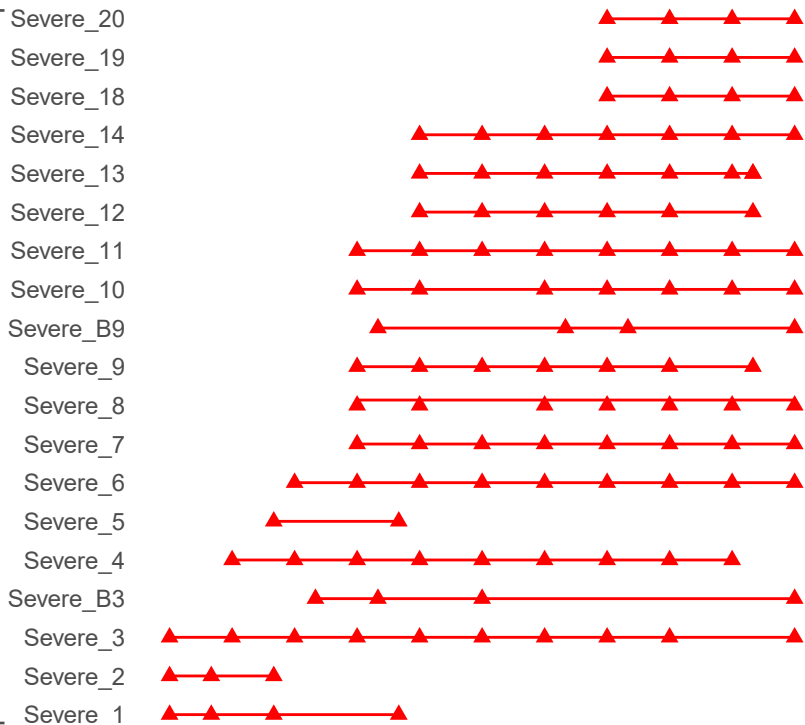

Type

nonSevere

Severe

Mild

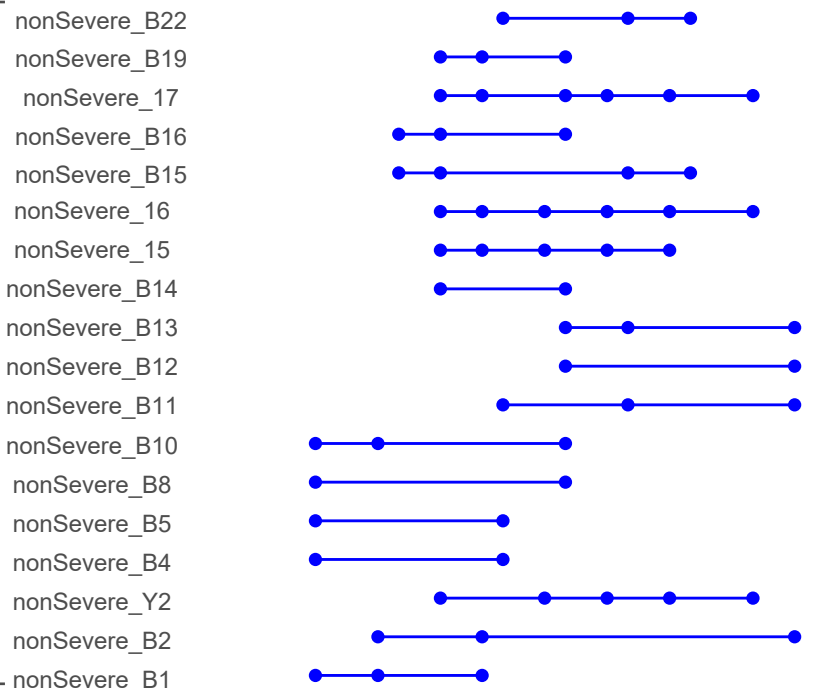

Day

date

Supplement: Supplementary file 6 — Supplementary file6 Fig. S1. Blood sample collection timepoints of COVID-19 patients in cohort 1 (PDF 192 KB) [file 438_2023_2014_MOESM6_ESM.pdf]

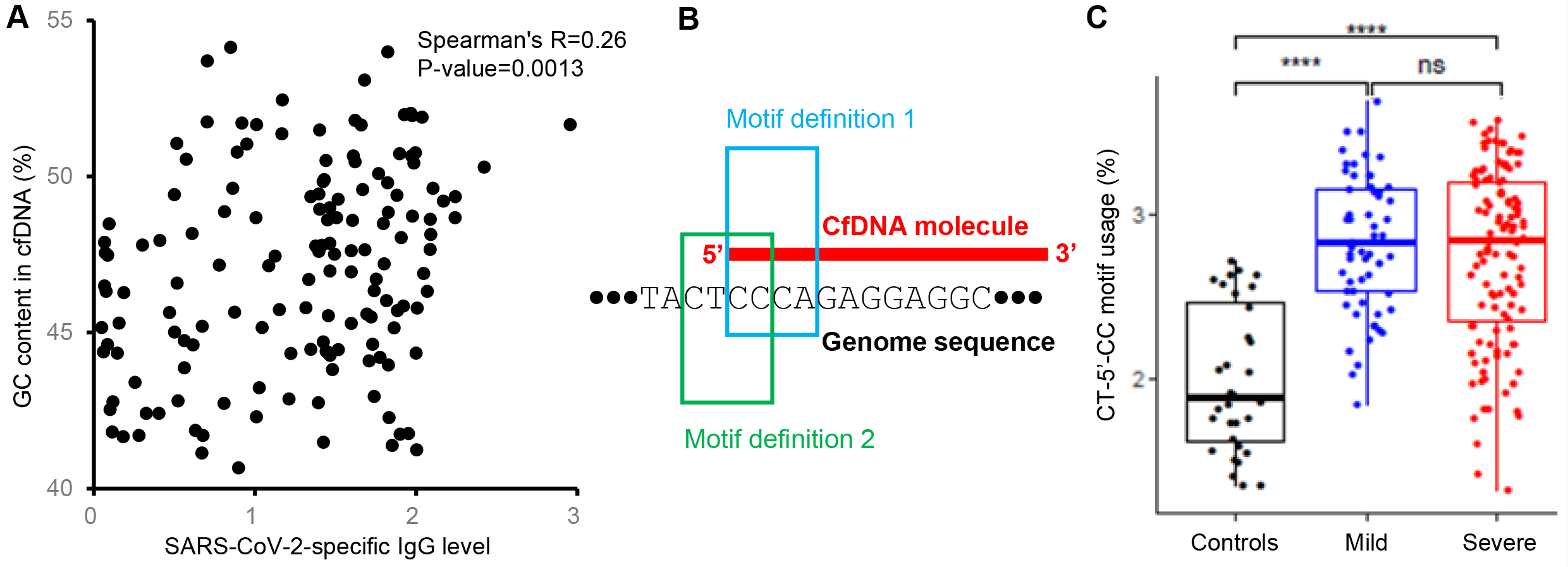

Supplement: Supplementary file 7 — Supplementary file7 Fig. S2. CfDNA characteristics in COVID-19 patients. (a) correlation between GC content in cfDNA of COVID-19 patients and SARS-CoV-2-specific IgG level (measured as OD values) in the corresponding blood sample; (b) definition of cfDNA end motifs used in this study; (c) Proportion of reads with CT-5′-CC end motif between controls and COVID-19 patients in cohort 1 and (d) cohort 2; (e) Clustering result of all the cfDNA samples in cohort 1 based on multiple characteristics (yield, GC content, size pattern, and 5′-CCCA end motif usage) (TIF 358 KB) [file 438_2023_2014_MOESM7_ESM.tif]

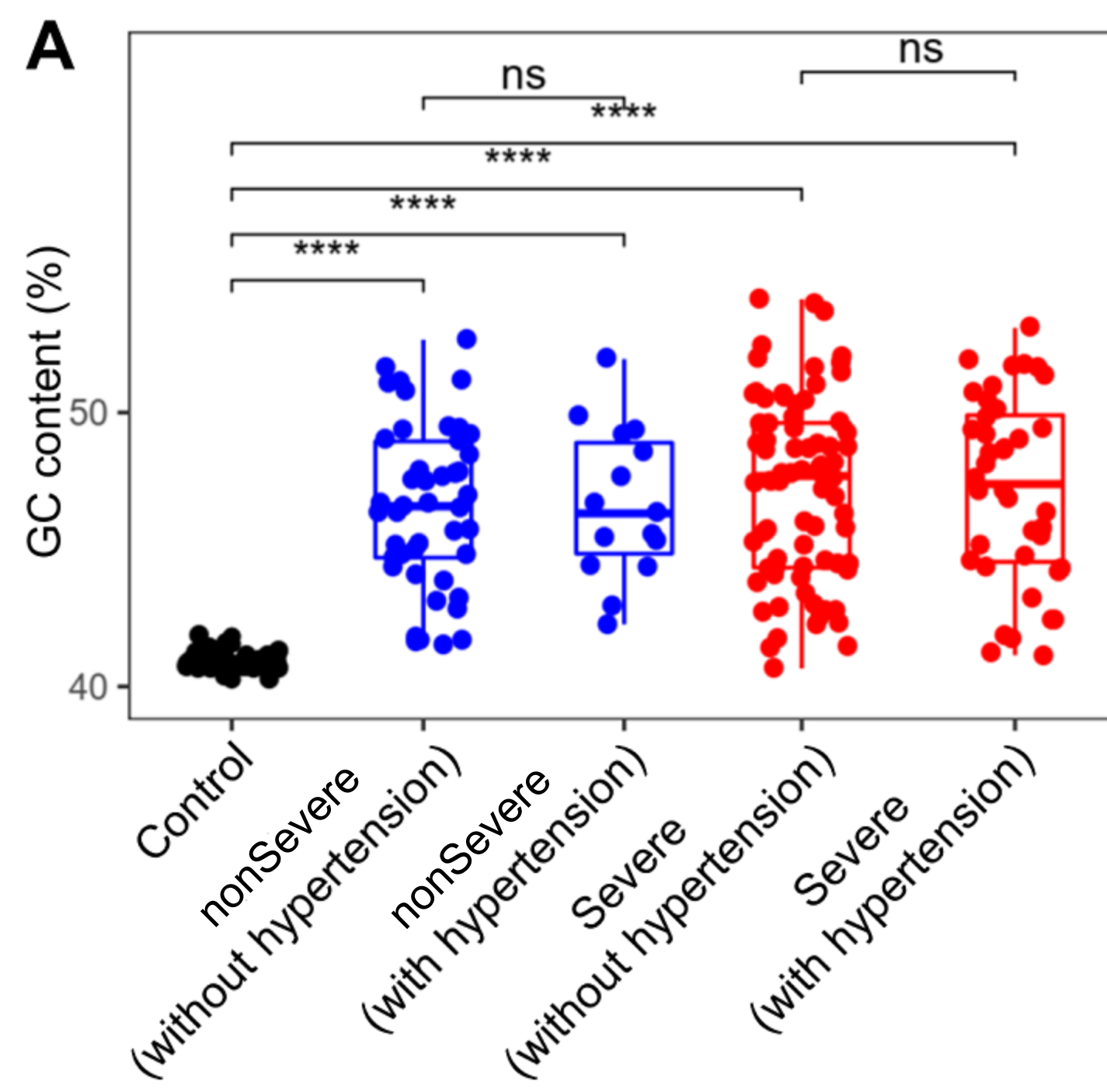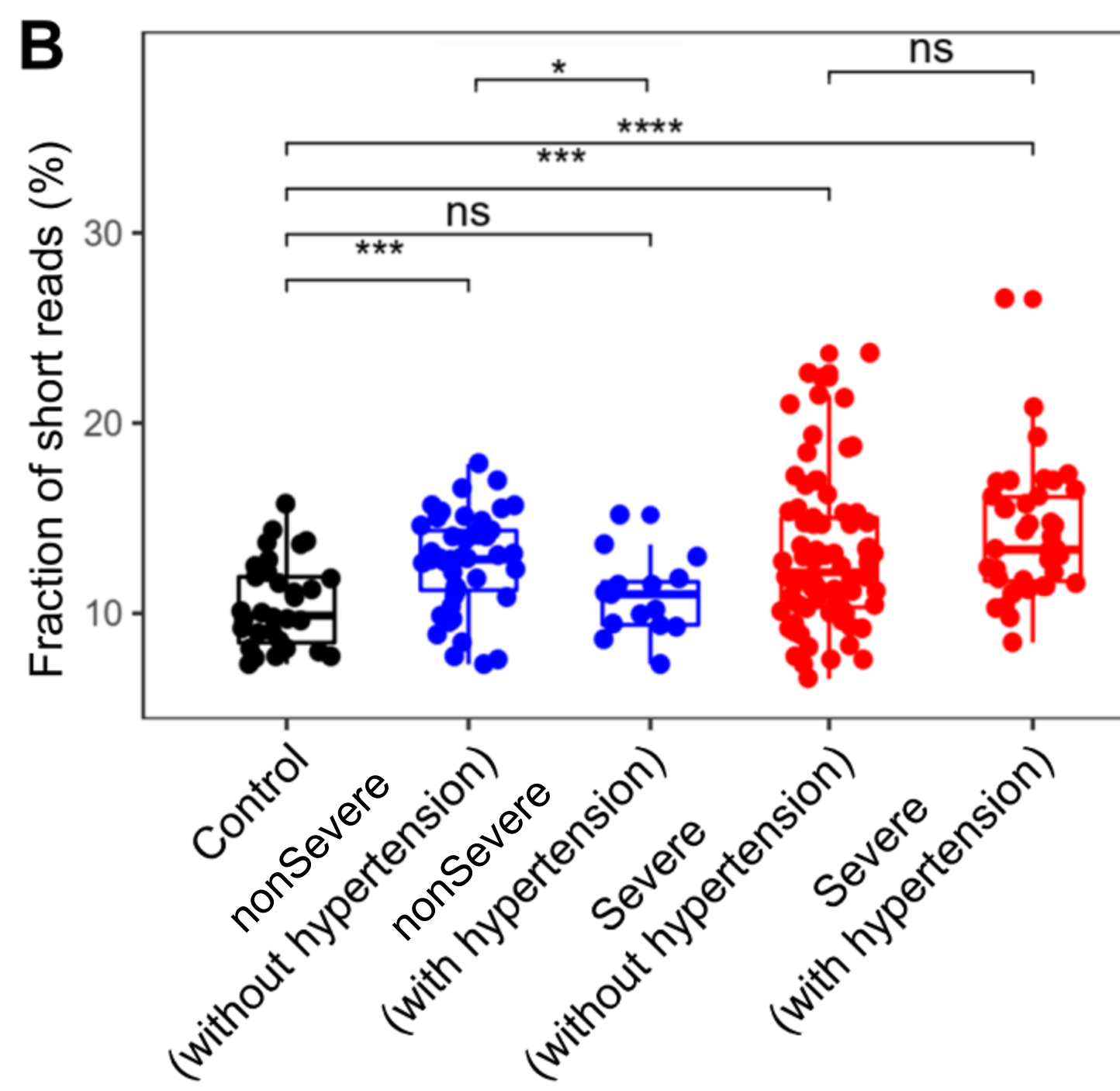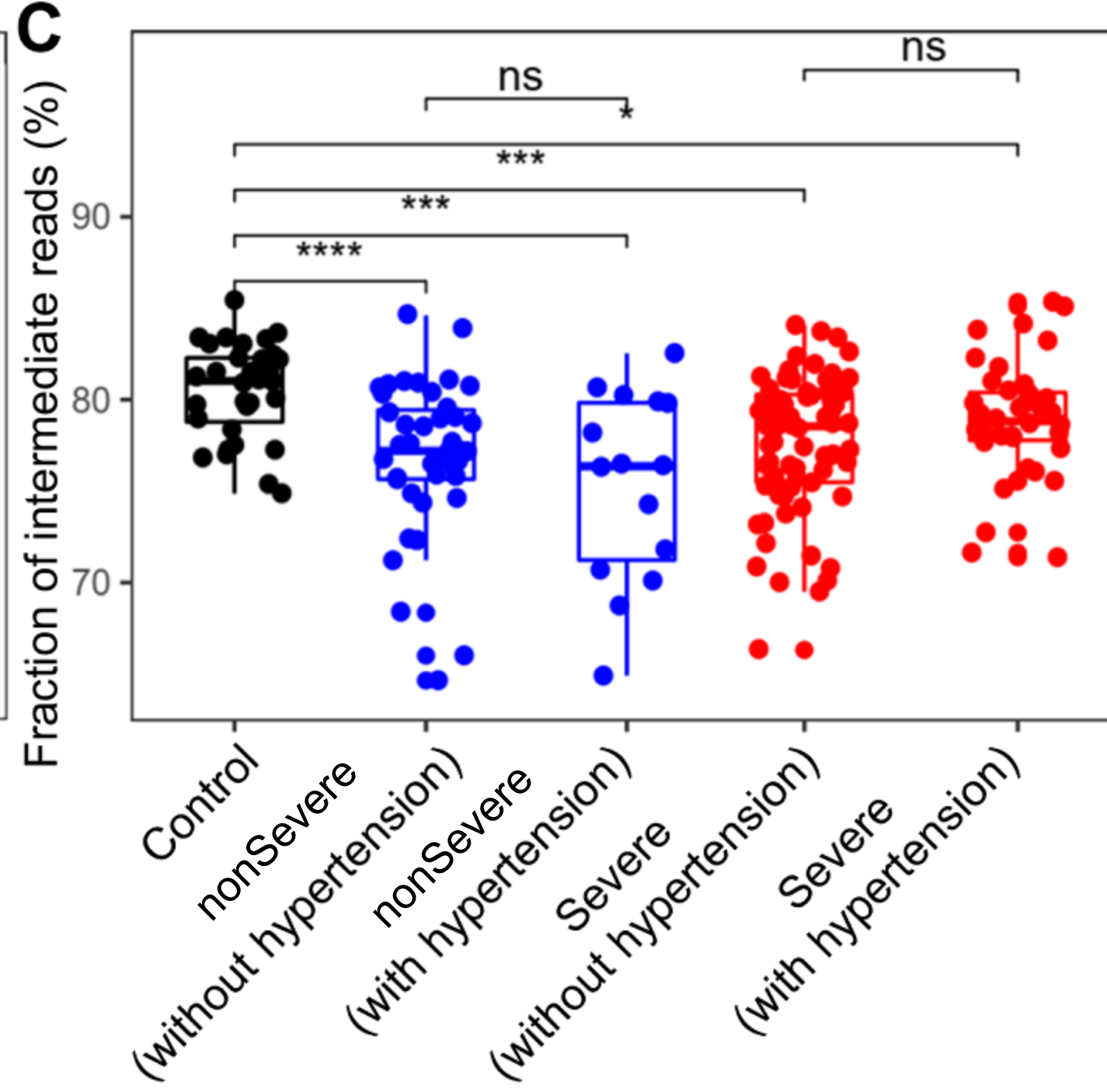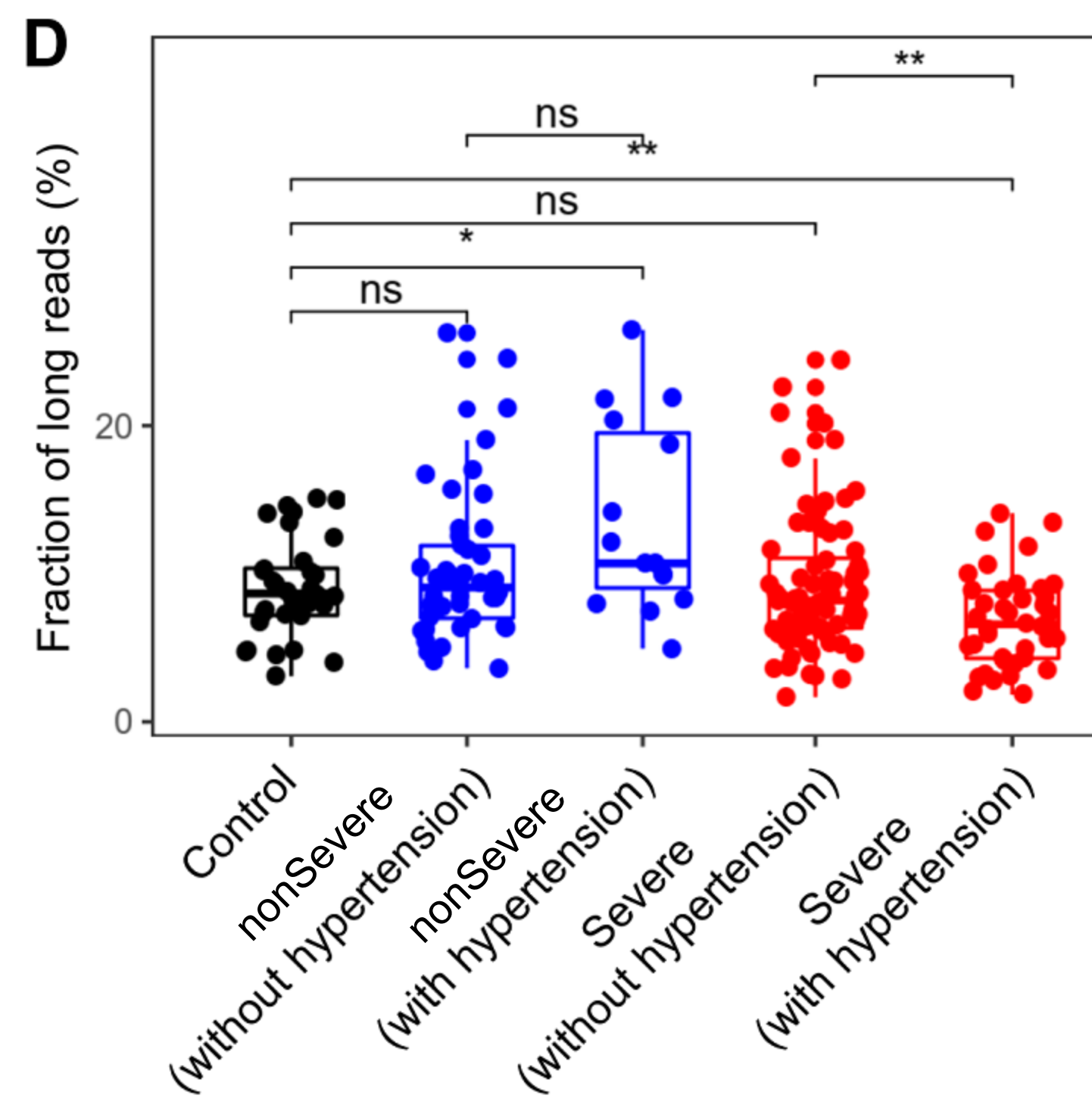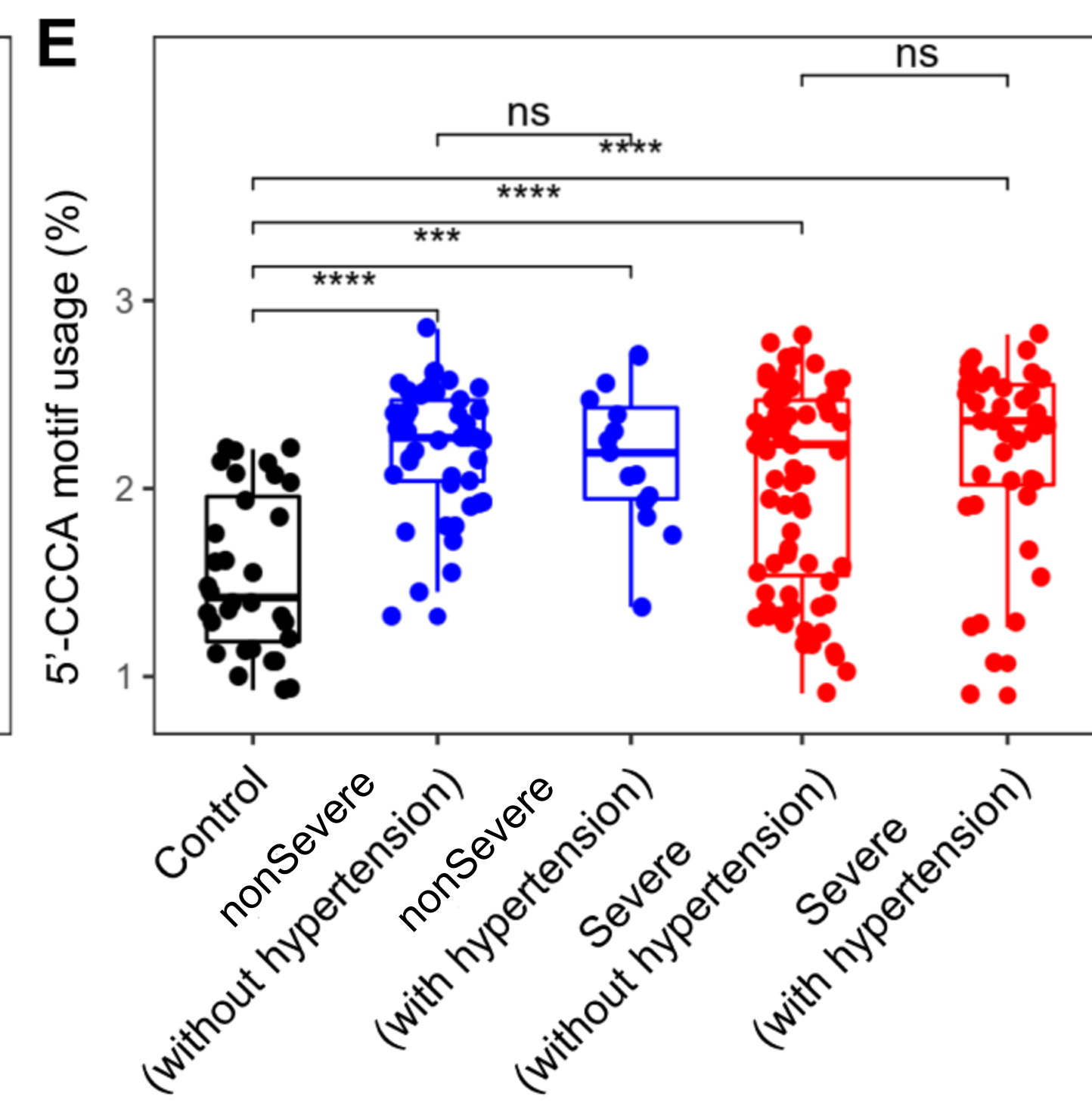

Supplement: Supplementary file 8 — Supplementary file8 Fig. S3. Dynamics of cfDNA characteristics during treatment of COVID-19 patients. (a-b) cfDNA concentration and CT-5′-CC end motif usage at the first timepoint versus the last timepoint in cohort 1; (c-d) cfDNA concentration and CT-5′-CC end motif usage at the first timepoint versus the last timepoint in cohort 2. For each panel, the 5 columns represent controls, first and last timepoints for nonSevere (blue) and severe (red) patients, respectively (PDF 1067 KB) [file 438_2023_2014_MOESM8_ESM.pdf]

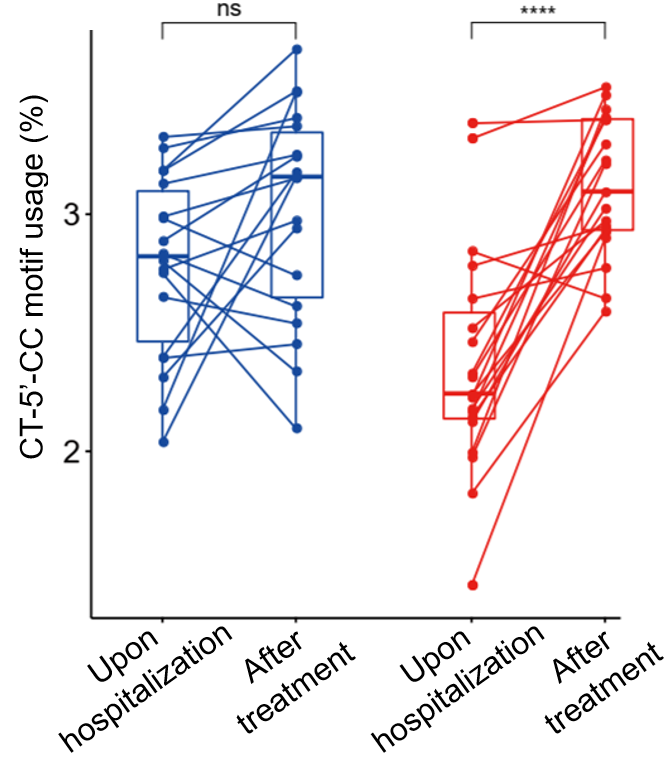

Supplement: Supplementary file 9 — Supplementary file9 Fig. S4. Time-series analysis of immunoglobulin levels and cfDNA characteristics for all COVID-19 patients in cohort 1. SARS-CoV-2-specific immunoglobulin levels are missing in some samples and are shown in blank. CfDNA concentration is measured as ng/ml; orange and green lines stand for proportion of short and long fragments, respectively; purple and blue lines stand for CT-5′-CC and 5′-CCCA end motif usages, respectively (TIF 273 KB) [file 438_2023_2014_MOESM9_ESM.tif]

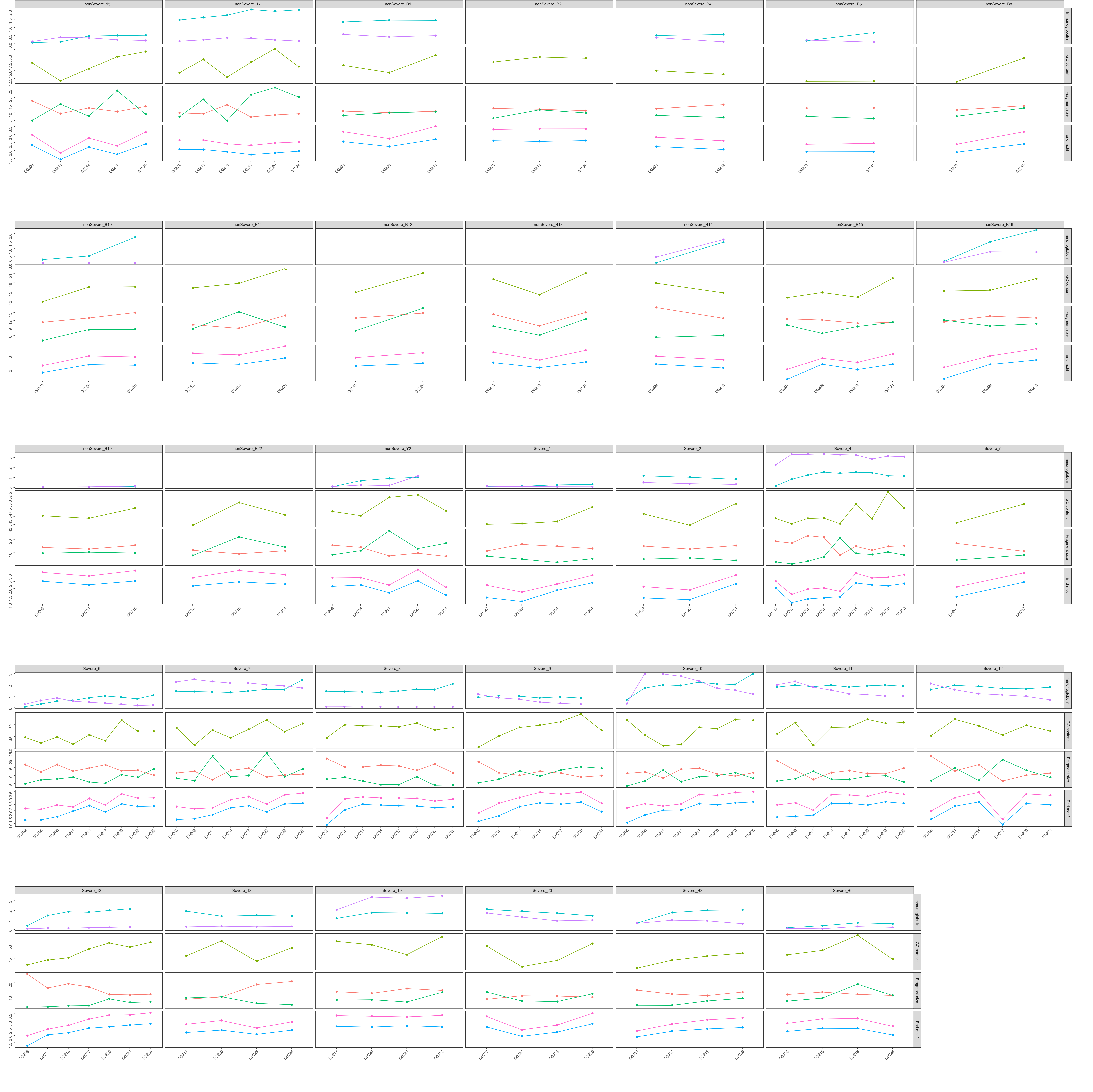

Supplement: Supplementary file 10 — Supplementary file10 Fig. S5. CfDNA coverage signal around tissue-specific open chromatin regions in multiple timepoints during treatment for COVID-19 patients in corhort 1 (PDF 662 KB) [file 438_2023_2014_MOESM10_ESM.pdf]

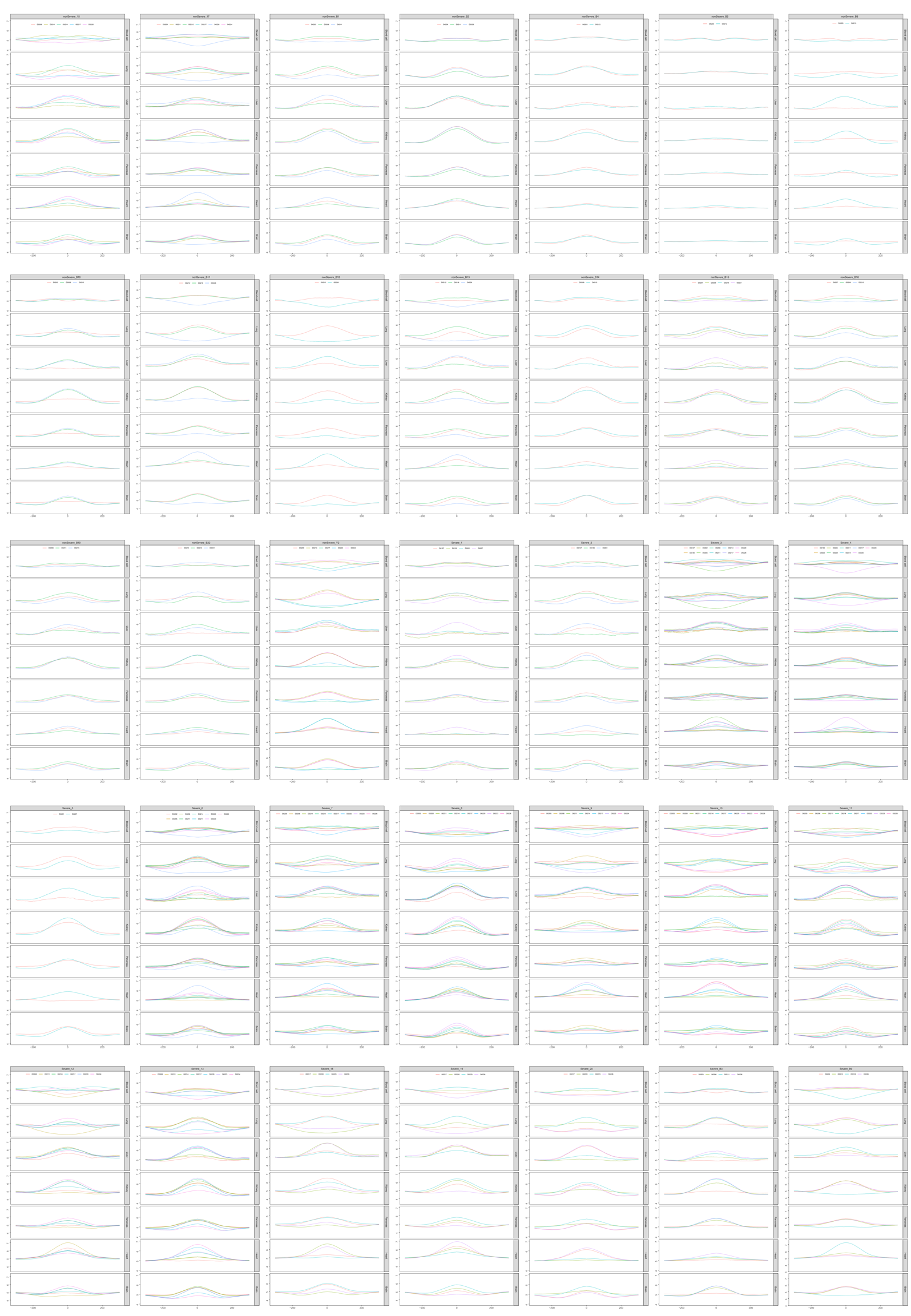

Supplement: Supplementary file 11 — Supplementary file11 Fig. S6. CfDNA coverage signal around tissue-specific open chromatin regions for COVID-19 patients in cohort 2 (PDF 14562 KB) [file 438_2023_2014_MOESM11_ESM.pdf]

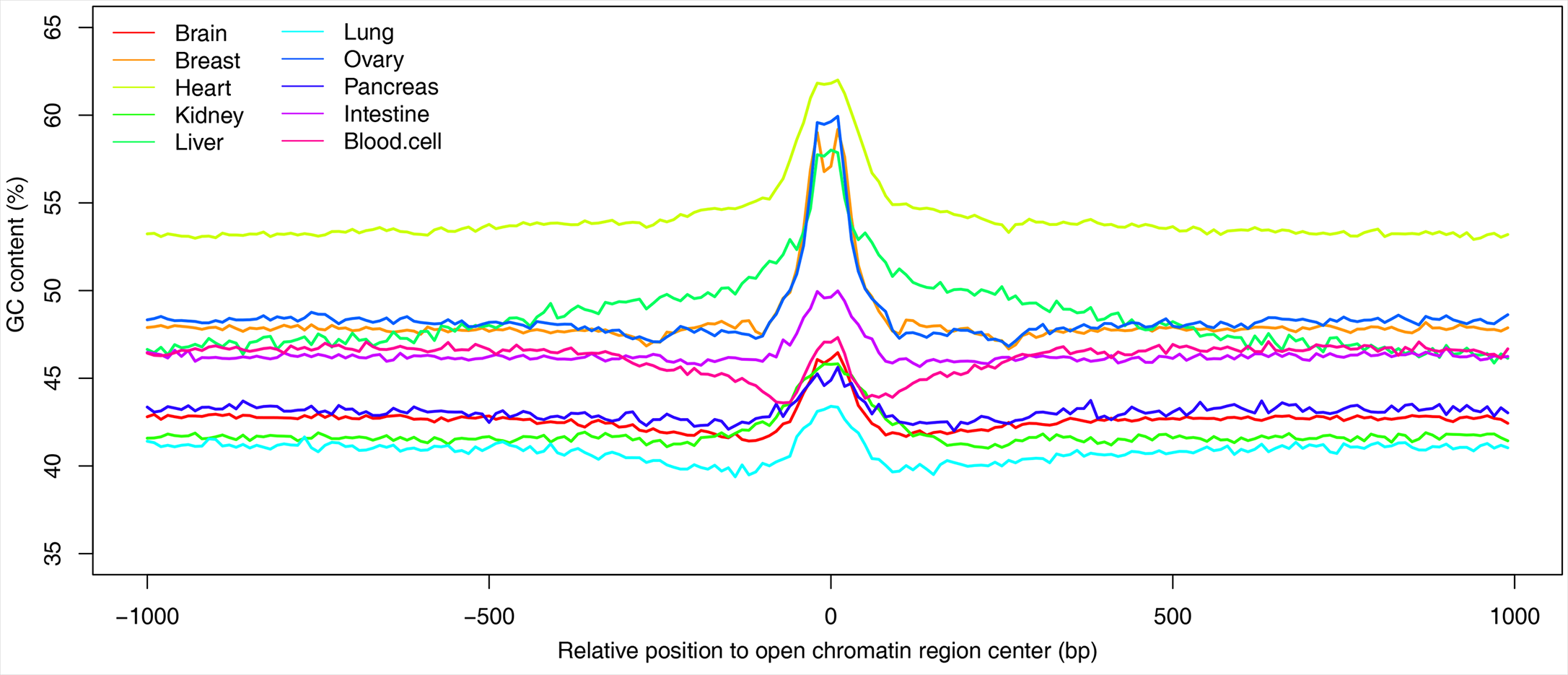

Supplement: Supplementary file 12 — Supplementary file12 Fig. S7. GC content around tissue-specific open chromatin regions (TIF 447 KB) [file 438_2023_2014_MOESM12_ESM.tif]

OCF for Lungs

25  
0  
-25

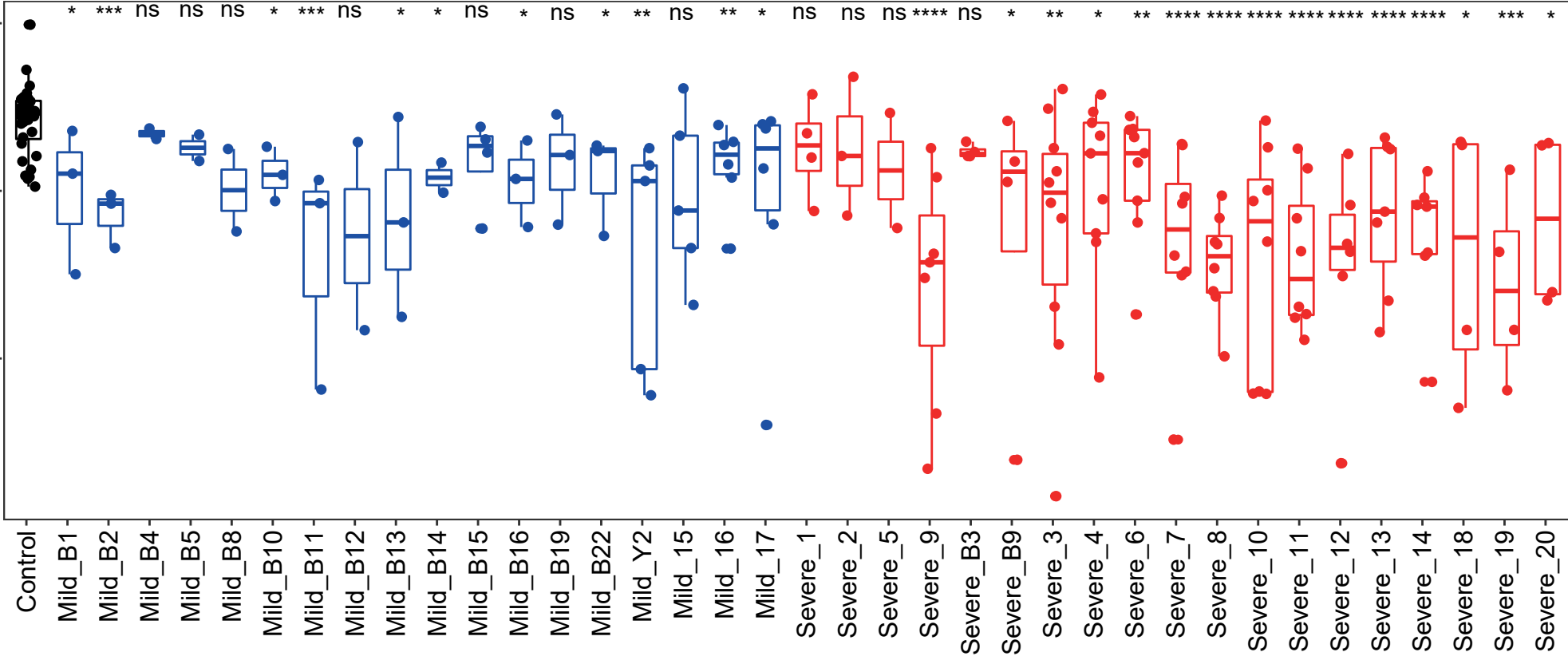

OCF for Liver

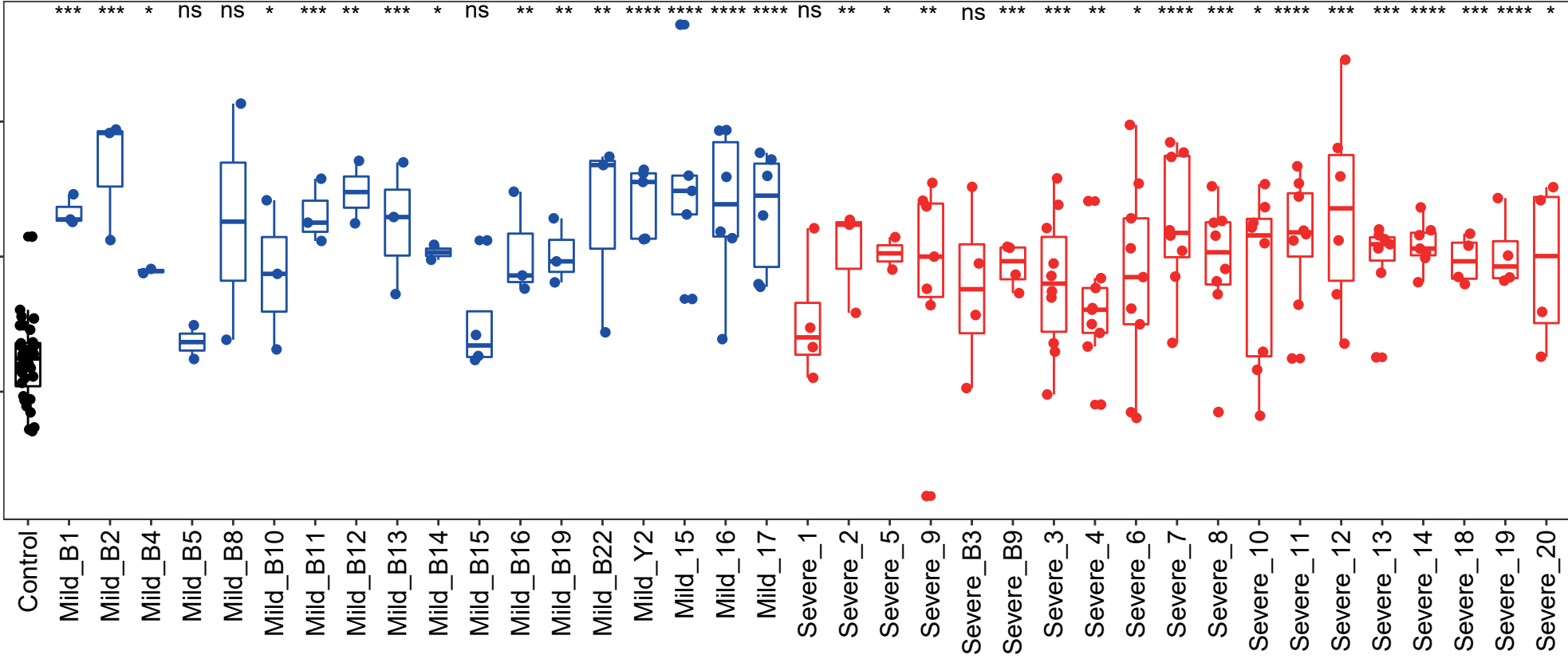

OCF for Kidneys

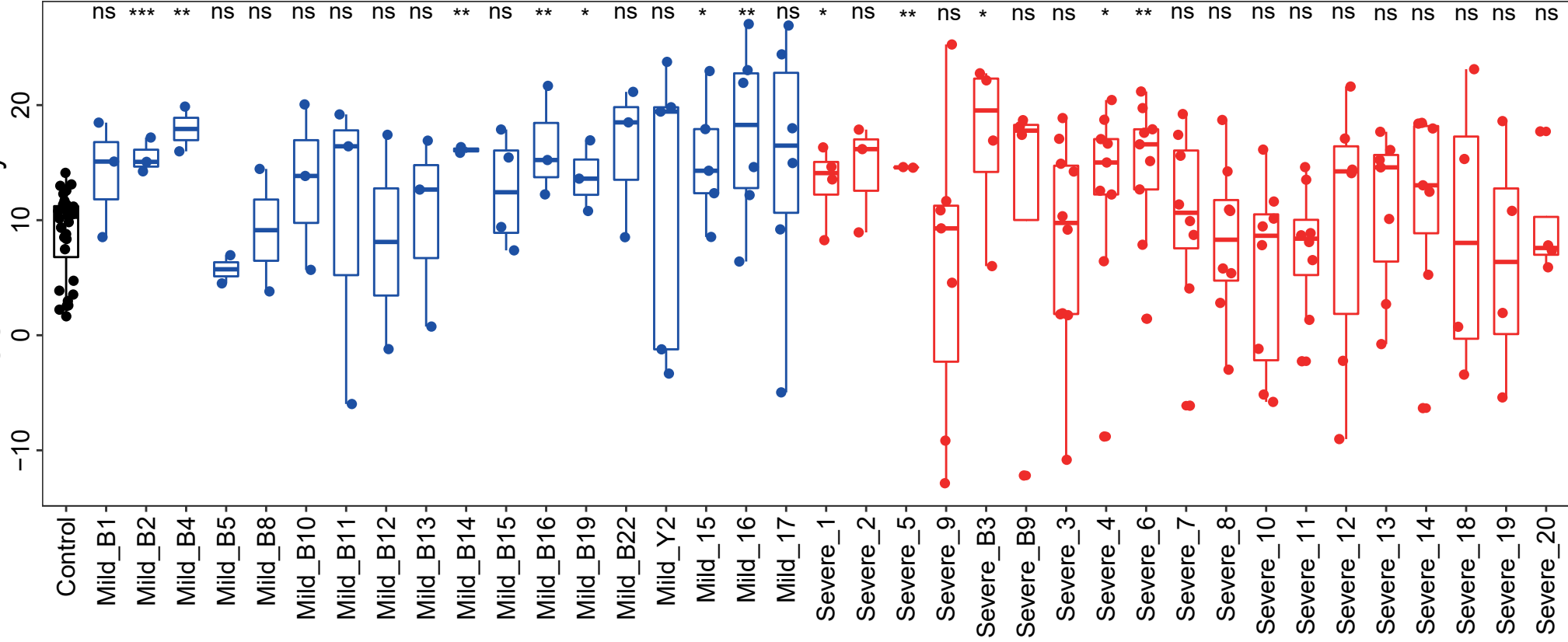

OCF for Heart

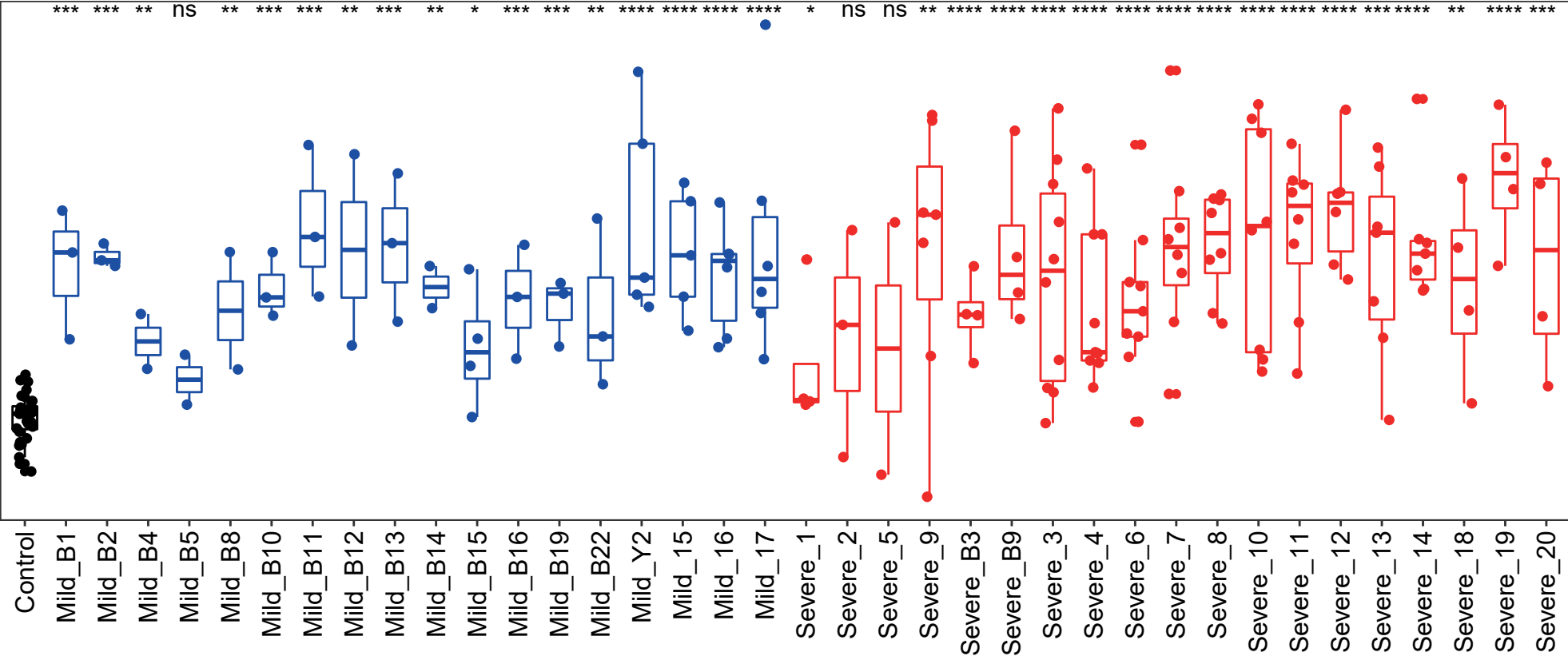

OCF for Pancreas

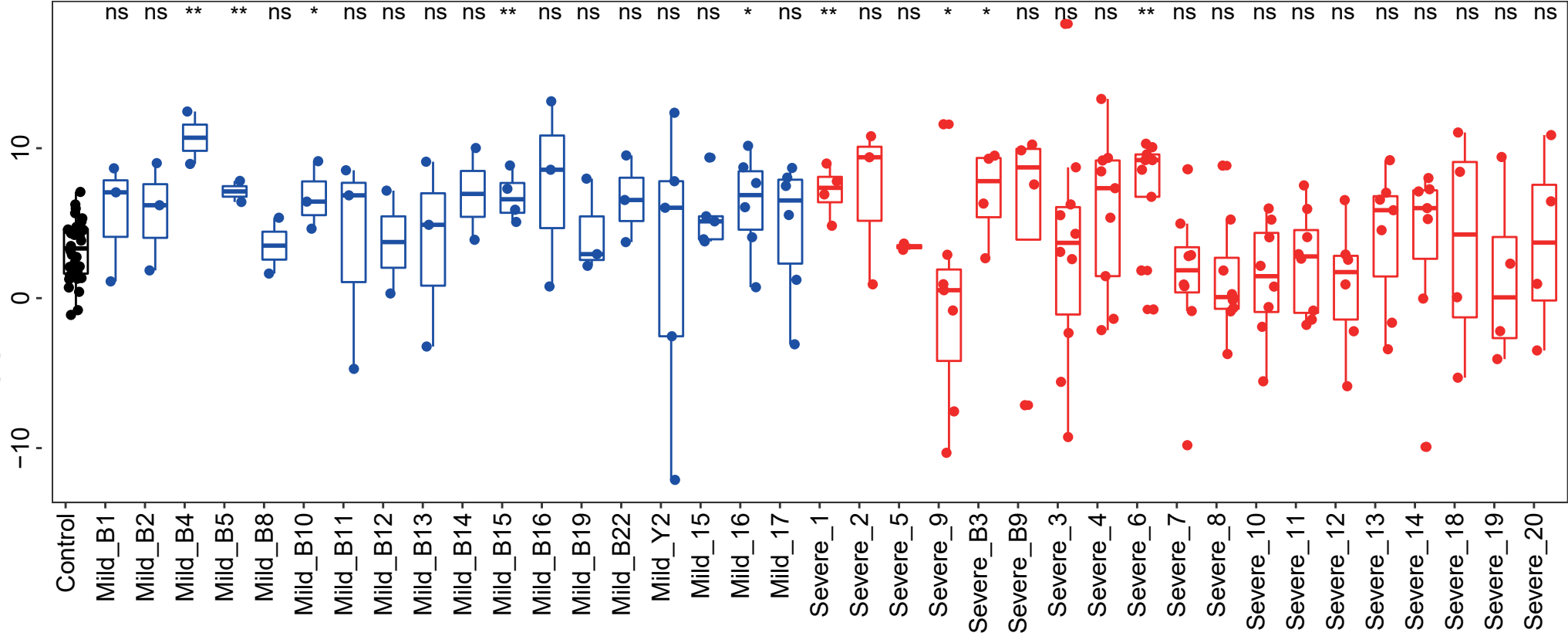

OCF for Brain

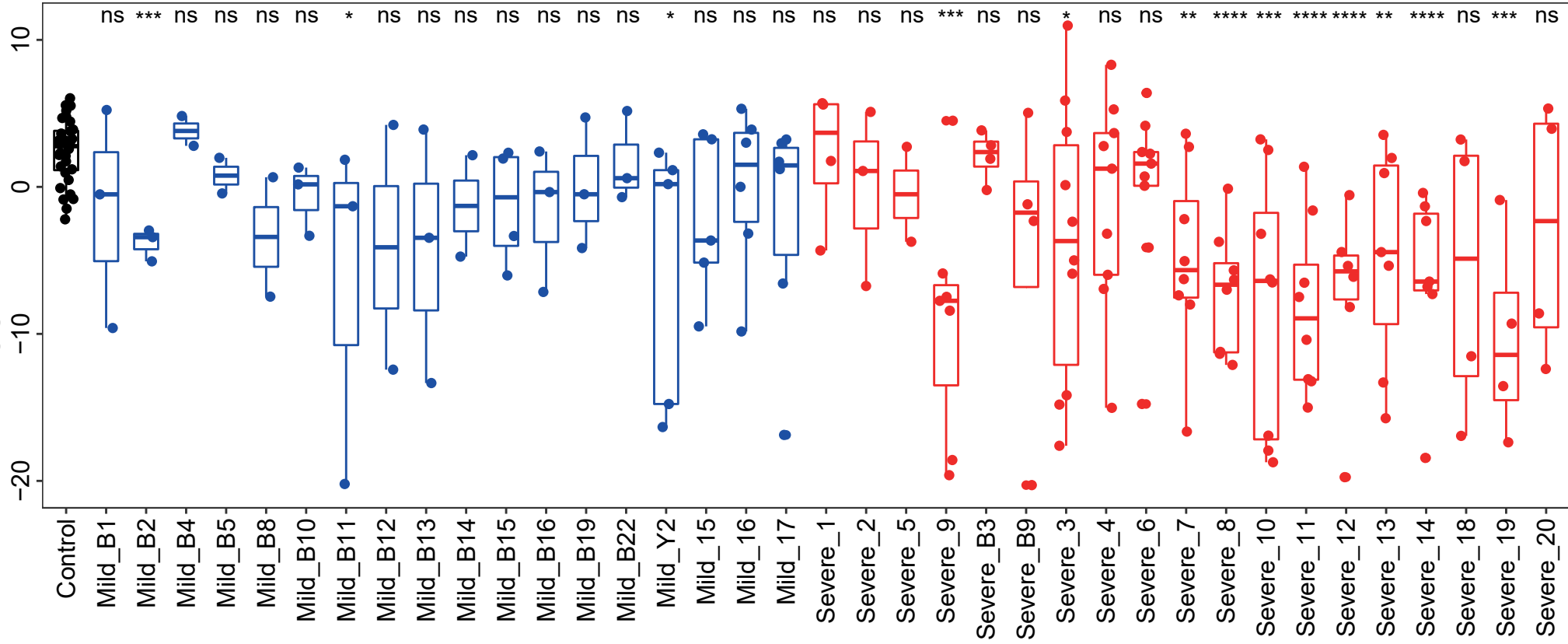

Supplement: Supplementary file 13 — Supplementary file13 Fig. S8. Summary of predicted tissue injuries in all COVID-19 patients in cohort 2 based on orientation-aware cfDNA fragmentation pattern analysis. For each case, tissue injuries of lungs, liver, kidneys, pancreas, heart, and brain are predicted. Clinical diagnoses were not available for all the patients in this cohort (PDF 281 KB) [file 438_2023_2014_MOESM13_ESM.pdf]
